# Effect of Biodegradable Hydrophilic and Hydrophobic Emulsifiers on the Oleogels Containing Sunflower Wax and Sunflower Oil

**DOI:** 10.3390/gels7030133

**Published:** 2021-09-07

**Authors:** Deepti Bharti, Doman Kim, Miguel Angelo Cerqueira, Biswaranjan Mohanty, SK Habibullah, Indranil Banerjee, Kunal Pal

**Affiliations:** 1 Department of Biotechnology and Medical Engineering, National Institute of Technology Rourkela, Rourkela 769008, India; deeptibharti94@gmail.com; 2 Department of International Agricultural Technology & Institute of Green BioScience and Technology, Seoul National University, Seoul 151742, Gwangwon-do, Korea; kimdm@snu.ac.kr; 3 International Iberian Nanotechnology Laboratory, Av. Mestre José Veiga s/n, 4715-330 Braga, Portugal; miguel.cerqueira@inl.int; 4 Department of Pharmaceutics, Institute of Pharmacy and Technology, Salipur, Cuttack 754202, India; biswaranjanm5@gmail.com (B.M.); skhabibullah.lucky@gmail.com (S.H.); 5 Department of Bioscience & Bioengineering, Indian Institute of Technology, Jodhpur 342037, India; indraniliit@gmail.com

**Keywords:** sunflower wax, sunflower oil, oleogels, emulsifier, fat crystal behavior

## Abstract

The use of an appropriate oleogelator in the structuring of vegetable oil is a crucial point of consideration. Sunflower wax (SFW) is used as an oleogelator and displays an excellent potential to bind vegetable oils. The current study aimed to look for the effects of hydrophobic (SPAN-80) and hydrophilic (TWEEN-80) emulsifiers on the oleogels prepared using SFW and sunflower oil (SO). The biodegradability and all formulations showed globular crystals on their surface that varied in size and number. Wax ester, being the most abundant component of SFW, was found to produce fibrous and needle-like entanglements capable of binding more than 99% of SO. The formulations containing 3 mg of liquid emulsifiers in 20 g of oleogels showed better mechanical properties such as spreadability and lower firmness than the other tested concentrations. Although the FTIR spectra of all the formulations were similar, which indicated not much variation in the molecular interactions, XRD diffractograms confirmed the presence of β′ form of fat crystals. Further, the mentioned formulations also showed larger average crystallite sizes, which was supported by slow gelation kinetics. A characteristic melting point (T_m_~60 °C) of triglyceride was visualized through DSC thermograms. However, a higher melting point in the case of few formulations suggests the possibility of even a stable β polymorph. The formed oleogels indicated the significant contribution of diffusion for curcumin release. Altogether, the use of SFW and SO oleogels with modified properties using biodegradable emulsifiers can be beneficial in replacing saturated fats and fat-derived products.

## 1. Introduction

Solid fats/triglycerides are common in the bakery industry due to their unique capacity to provide texture, flavor, and aroma to baked products. Saturated and trans-fats are usually rich in solid fat. As the apprehension regarding the use of *trans* and saturated fats on human health is rising worldwide, the World Health Organization (WHO) and Food and Agriculture Organization (FAO) are endorsing to increase the amount of mono and polyunsaturated fatty acids in food products [1]. United States Food and Drug Administration (US-FDA) in 2015 recognized trans-fats as unsafe for human consumption. Accordingly, the use of trans-fats were wholly banned for use in food products [2]. The search for solid fat replacers bought scientists closer to the concept of oleogels. Oleogels have received remarkable achievements in medicine, cosmetics, food, and biomaterials [3].

The process of oleogel formation is commonly known as “oleogelation”. Oleogelation is a method of oil structuring without modifying the chemical composition of the oils. The structuring process allows converting vegetable oils that are rich in unsaturated fats into textured products. Such structuring is possible using oleogelators, which either forms self-assembled network structures within the oil phase or create crystalline networks to structure the oil [4]. In vegetable oils, various gelators such as waxes, ceramides, monoacylglycerides, and sugar alcohol-based oleogelators have been explored to structure oils. Low molecular weight organogelators can induce gelation of oils at significantly lower concentrations. The gelled structures exhibit rheological properties closer to solid materials at an optimum temperature [5]. Natural waxes have shown promising outcomes due to their easy accessibility, little cost, and a great extent of structuring. Waxes contain long-chain hydrocarbons, fatty alcohols, wax esters, and fatty acids [6]. Usually, the melting of the wax at a higher temperature in combination with the oil, followed by a cooling step below the crystallization temperature, results in oleogels formation [7]. One such high melting gelator is sunflower wax (SFW), which is prepared from the winterization of sunflower oil (SO). SFW has a melting temperature ranging from 74 to 80 °C. A significant portion of the SFW is longer chains of wax esters. The food products made from formulations containing a lower amount of waxes are appreciable. This helps to avoid the waxy coating of the mouth during the consumption of food products [4]. Additionally, the oleogels developed using a lower amount of plant-derived waxes, as oleogelator, are highly stable and show excellent strength [8].

Previous studies have suggested that SFW not only has good oil binding capacity but can also form oleogels at a shallow gelator concentration (<10 wt.%) [9]. This can be attributed to esters in SFW that exhibit platelet crystal morphology to form dense networks of high strength [10]. Oleogels prepared from the structuring of soybean oil using SFW were used as a solid fat replacer in margarine and spreads [11]. Regarding the vegetable oil used for structuring, SO is the most commonly used edible oil with enormous health benefits [12]. They usually have a low level of saturated fatty acids and a high amount of poly and monounsaturated fatty acids. Besides, SO is also enriched with Vitamin A and Vitamin E, which have antioxidant properties. Altogether, SO is known to improve immune system functioning, cardiac function, and skin health. Recently, wax-based SO oleogels have been attempted to replace the shortening in cakes without hampering the quality [13]. The oleogels were found to form nutritionally rich cakes with a significantly reduced sum of saturated fatty acids.

The gelation is often affected by solvent quality, crystal memory, temperature, shearing force, and additives [14]. The synergistic effect on oleogels due to the interaction of gelator and additives such as emulsifiers has not been suitably discovered. Since the gelling mechanism is highly dependent upon the gelator-oil and gelator-gelator concentration, thus the addition of emulsifiers can influence the gelation kinetics and physical properties of prepared oleogel. This can be achieved by altering the gelator-oil interaction that, in response, can affect the oil mobility in the gel network. Among many available emulsifiers, SPAN and TWEEN are commonly used non-ionic surfactants in food and pharmaceutical applications. These emulsifiers have been reported to modify the nucleation and crystal growth of fats [15]. The addition of liquid emulsifiers such as hydrophilic TWEEN-80 and hydrophobic SPAN-80 have shown alteration in the appearance, thermal, crystallization, and texture properties of oleogels prepared from stearic acid and soybean oil [16]. SPAN-80 (Hydrophilic and lipophilic balance, HLB = 4.3) and TWEEN-80 (HLB = 15) are two commonly used biodegradable emulsifiers in food industries [17]. As displayed by the HLB values, emulsifiers with different hydrophobicity can alter the rate of fat crystallization [18]. They can also influence the properties of polymeric transitions taking place during crystallization. The used emulsifiers are approved by US-FDA and are safe to use as food additives [19].

As discussed before, the oleogel formation depends on the interaction ratio of the solvent-gelator among the gelator molecules. The various properties of formulated oleogels are studied in terms of solubility, molecular interaction, structural arrangement, thermal behavior, and mechanical stability. In the present study, wax oleogels containing liquid emulsifiers were prepared by the heating-and-cooling method. The physicochemical characteristics of the waxes, which their chemical compositions can explain, make them suitable as a gelator. The current study attempts to understand the effect of the increasing amount of non-ionic hydrophilic (TWEEN-80) and hydrophobic (SPAN-80) emulsifiers on the color, binding capacity, microarchitecture, mechanical, molecular arrangement, crystallization, and thermal properties of an oleogel of SO containing 5% (*w*/*w*) of SFW. Further, the release of curcumin from oleogels was also tested to understand the effect of emulsifiers in modulating the release of the bioactive compound. Therefore, it is worthwhile to look for the effectiveness of biodegradable emulsifiers in the wax oleogels having the potential to act as a solid fat replacer.

## 2. Results

### 2.1. Visual Appearance and Oil Binding Capacity

**Figure 1:**
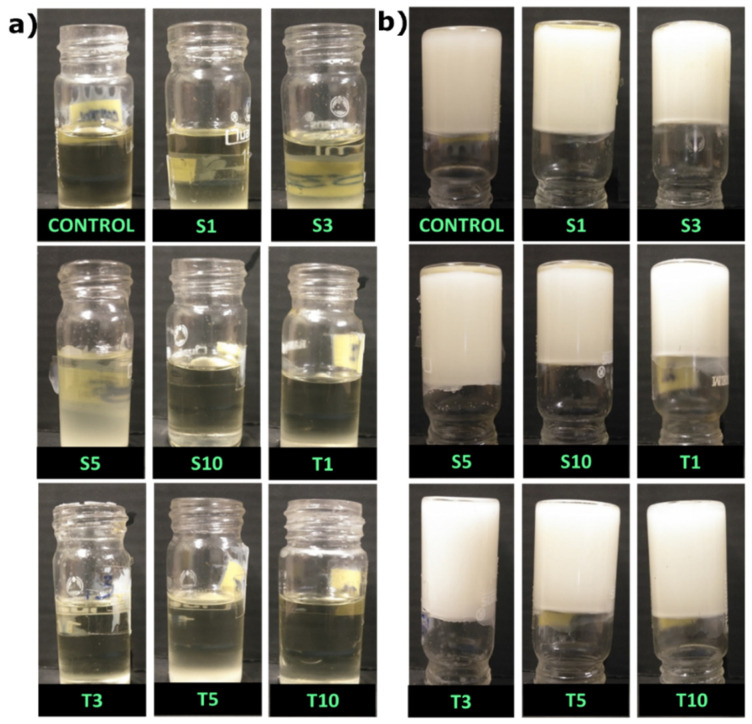
(**a**) Homogenous mixture of formulations; (**b**) inverted tube method. [S1, S3, S5 and S10: 1, 3, 5 and 10 mg of SPAN-80 in oleogels of SO containing 5% (*w*/*w*) of SFW; T1, T3, T5 and T10: 1, 3, 5 and 10 mg of TWEEN-80 in oleogels of SO containing 5% (*w*/*w*) of SFW].

The SO, SFW, and liquid emulsifier mixture were heated at 80 °C in the water bath to form a clear homogenous solution (Figure 1a). Oleogels were formed after placing them for 30 min in the thermal cabinet, as discussed above (Figure 1b). This was followed by inverting the sample bottle and maintaining it at room temperature for ~30 min to observe the visual appearance and confirm the gel formation. All the formulations used in our study could form compact structures that were self-standing, which is similar to previously reported work [19]. The visual appearance did not significantly differ among all the formulations. Though SPAN-80 formulation appeared identical to the control, the inclusion of TWEEN-80 in the oleogels may slightly improve the formulations’ whiteness. The formed oleogels were smooth with easier spreadability when touched with hands.

The oleogels were also evaluated for their capacity to bind oil. The SFW was proven to be efficient in binding the SO, evident from the %OBC values. The %OBC was greater than 99% in all the cases. Previous work has reported a stronger mechanical strength of oleogels because of the higher OBC [20]. The addition of both the emulsifiers at the selected amount did not significantly affect the oil binding capacity of the oleoegels. Interestingly, the crystalline phase formed from a 5% wax concentration was sufficient to develop a network to hold sunflower oil into the oleogel. A similar wax concentration has been reported to structure soyabean oil, which is commonly used to replace saturated fats in food products [10]. Waxes containing a long chain of alkanes and esters are much more efficient in vegetable oil gel than waxes made of short-chain alkanes [21]. Many natural waxes such as carnauba wax, candelilla wax, and beeswax, etc., have been reported as an efficient oleogelator because of their ability to crystallize the vegetable oil at lower concentrations [22]. The lower wax concentration for formulating oleogels is attributed to the presence of long-chain esters in the SFW. The emulsifiers in the proposed concentrations did not significantly affect the oil binding capacity of the oleogels.

### 2.2. Colorimetric Analysis

The L*, a*, and b* or CIE Lab is defined by Commission Internationale de l’Eclairag and is widely accepted for color measurement in food products [23]. According to this, all the colors are a combination of red, green, and blue, whose receptors are present in the human eye. The luminance (L*) is referred to as the lightness component, ranging from 0 to 100. Since all our formulations had shown an L* value close to 100, it was inferred that oleogels were substantially luminous. This can be possible due to the presence of smaller fat structures on the surface of oleogels that could reflect most of the light [24].

**Figure 2:**
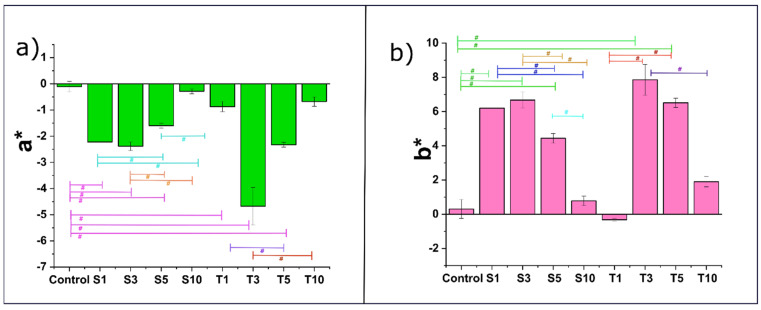
Colour parameters of the oleogels: (**a**) a* values; and (**b**) b* values. The values in the graph are denoted as the mean of the triplicate ± standard deviation (*p* < 0.05) The significantly different values are represented with symbol #. The details of abbreviations used are provided in Section 4.2 (Table 5).

Additionally, a* and b* (Figure 2) are the chromatic components that range from green to red and blue to yellow, respectively [25]. The values of these chromatic components range from −120 to +120 [25]. For the values of a* the range follows −ve (red) and +ve (green). For all the formulations, a* value was found negative. This gives an idea about the presence of a better fraction of green hue. Similar results have been reported with oleogels formed from SFW in the previous work [26]. The addition of emulsifiers has shown more shift towards green as compared to the control. The addition of SPAN-80 showed a subsequent increase from the control in the a* value till S3. However, the extent of greenness was similar in S1 and S3. On increasing the emulsifier content in S5, a significantly higher a* value from the control was observed. This value was considerably lower than the a* value of S1 and S3. At the maximum SPAN-80 content, the extent of the greenness of the formulation S10 was similar to the control. Further, the addition of TWEEN-80 showed a subsequent increase from control in the a* value till T5. However, till T5, the rise in the green hue of T5 was only significantly different from T1. Formulation T10 showed a similar a* value as the control, T1, and T5. However, the reduction in the a* of T10 from T3 was significant. Among the formulations, T3 showed the highest share of green, followed by S3.

Further, the b* value in all the formulations except T1 appeared positive. The b* values display −ve (blue) to +ve (yellow). Thus, a positive b* value indicates a more significant proportion of yellow. Among the SPAN-80 formulations, the b* value followed the same trend as that of a*. However, the addition of TWEEN-80 in T1 showed a similar b* value as the control. A significant increase in the b* value of T3 from control and T1 was observed. A further rise in the TWEEN-80 content in T5 displayed a substantial surge in the yellowness from the control and T1. On comparing T5 with T3, there was no difference in the b* values. At the highest emulsifier content in T10, the yellowness of the formulation appeared similar to the control, T1 and T5. However, the reduced b* value in T10 from T3 was significant. Similar to a* value, T3 has shown the highest share of yellow, followed by S3. The absolute colour difference (ΔE) is a numerical value obtained from L*, a*, and b* and is generally used to compare the samples with a specific standard (in our case, the control). The calculated values of ΔE are represented in Table S1.

### 2.3. Microscopic Analysis

#### 2.3.1. Surface Topography

**Figure 3:**
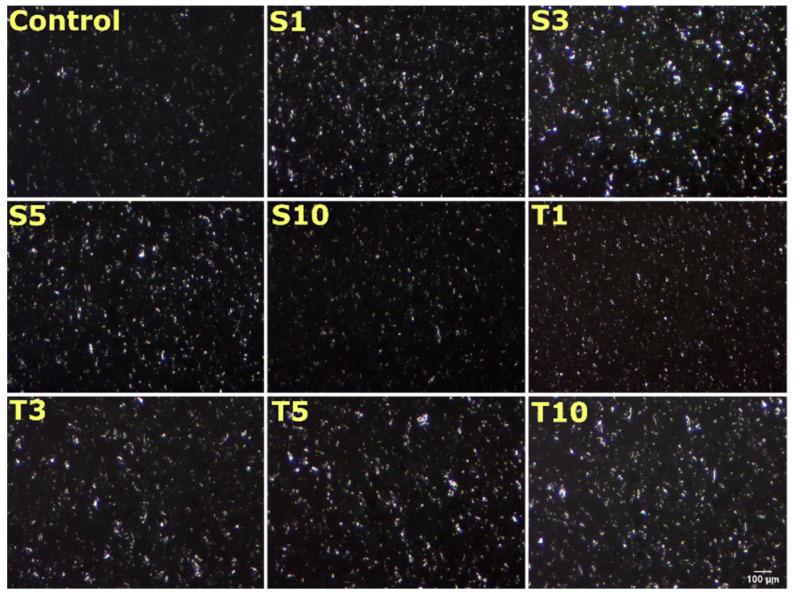
Surface topology images of oleogels representing the presence of globular structures. Scale bar: 100 µm. (**S1–S10**: Increasing amount of SPAN-80 content; **T1–T10**: Increasing amount of TWEEN-80 content). The details of abbreviations used are provided in Section 4.2 (Table 5).

The surface topology images display a uniform distribution of fat crystals in all the formed oleogels (Figure 3). Obtained topographs clearly show the semi-crystalline structure of oleogels, which has both crystalline and amorphous regions. The fat crystals appeared as globular structures. The fat crystals varied in number and size in all the formulations. For example, the inclusion of SPAN-80 increased the number and size of the crystals in S1 and S3 compared to control. The crystals appeared bright and were most prominent in S3. In S5, the size of fat crystals was reduced; however, S10 showed similar globular size and density to the control. Similarly, an increase in TWEEN-80 concentration in oleogels displayed a rise in crystal structure and distribution size. The increase in the size of the crystals can be due to the co-crystallization of these emulsifiers with triacylglycerol. The phenomenon of co-crystallization may have enhanced the crystal growth [27]. The overall surface topology appears as the dispersion of many small fat crystals, which is the desired property for food applications [28].

#### 2.3.2. Microstructure Analysis

**Figure 4:**
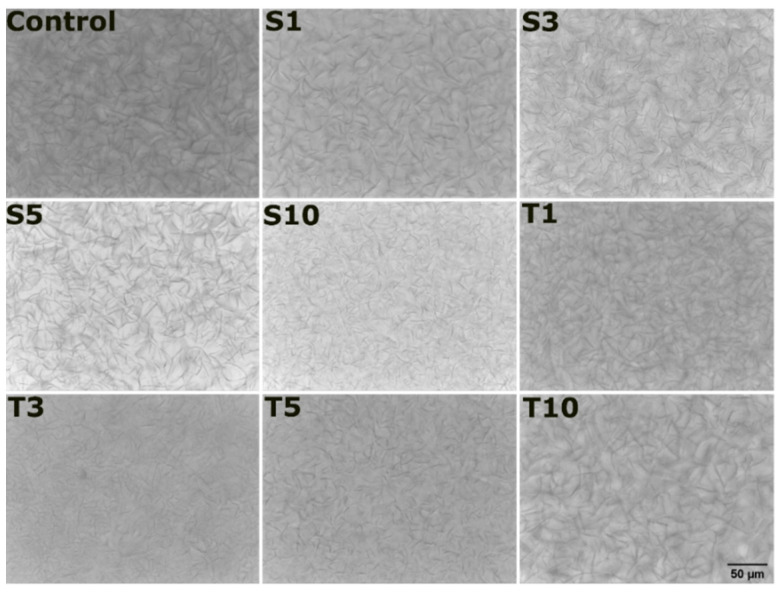
Bright-field micrographs of all the formulations. The details of abbreviations used are provided in Section 4.2 (Table 5).

The micrographs obtained using bright field and polarized light microscopes were used to visualize the network formed by fats in the oleogel. Since the formation of the wax-based oleogel depends upon the entrapment of the oil phase through wax crystals, looking at the morphology and polymorphism of these crystals is an interesting way to comprehend the physical properties of the oleogel. The bright-field micrographs showed a fibrous network (Figure 4) in oleogels following previously reported work [26]. The reason here can be the high content of wax esters that are present in the SFW. Waxes or any low molecular weight oleogelators are known to form a three-dimensional-fibrous network. This is usually possible by molecular self-assembly of gelator molecules, which is governed by hydrogen bonding, π–π stacking, van der Waals forces, and hydrophobic forces. The molecular units of waxes are primarily linear. Hence, the crystals either grows in 1-dimension or 2-dimensions, thereby forming needle or plate-like structures, respectively [29]. The appearance of the fibre-like crystals results from the unidirectional growth of crystals from a single nucleation point [30]. A continuous branching was observed from various points of the fibres, which is typical for physical gels [31]. The self-assembled fibrillar network often forms these branches through crystallographic mismatch branching (CMB). The mechanism involves nucleation on the surface of the parent fibre of a daughter fibre which appears as a fork [32]. The fibre architecture of S1 seemed to be similar to control in terms of fibre length, thickness, and branching. However, a slight increase in the emulsifier’s amount in S3 increased the fibre length and hyper-branching. A high aspect ratio (length to diameter ratio) of fibres and their network is efficient in entrapping most of the oil [33]. A further rise in the amount SPAN-80 in S5 and S10 reduced the fibre length and branching. Similar to S1, the inclusion of TWEEN-80 in T1 did not significantly alter the fat crystal network’s architecture compared to the control. However, the number of fibres, in this case, has increased slightly. The fibres appeared thinner in T3 and T5, which again became thicker in T10. Another interesting observation was regarding the hyper-branching of the observed fibres, which was prominent in T1. After that, the hyper-branching of the fat crystals was reduced with the increase in the TWEEN-80 till T5. Nevertheless, in T10, the fibres suddenly started appearing longer as well as being hyperbranched.

Among the two types of liquid emulsifiers selected for this study, the SPAN-80 formulation displayed denser hyper-branching compared to TWEEN-80 formulations. This supports better fractal characteristics of lipid crystals in the SPAN-80 included oleogels [34]. The term fractal is associated with multiple crystal fractions, where each fraction holds a similar function to that of the parent crystal. Fractal dimension correlates with gels displaying an abundant number of crystals that are homogeneously distributed. These are usually calculated through computational methods such as the box-counting method [35]. The fractal dimension is associated with better oil binding capacity, an overall increase in the area for oil adsorption, and a consequent decrease in the void size of the 3-dimensional network of fat crystals [36].

**Figure 5:**
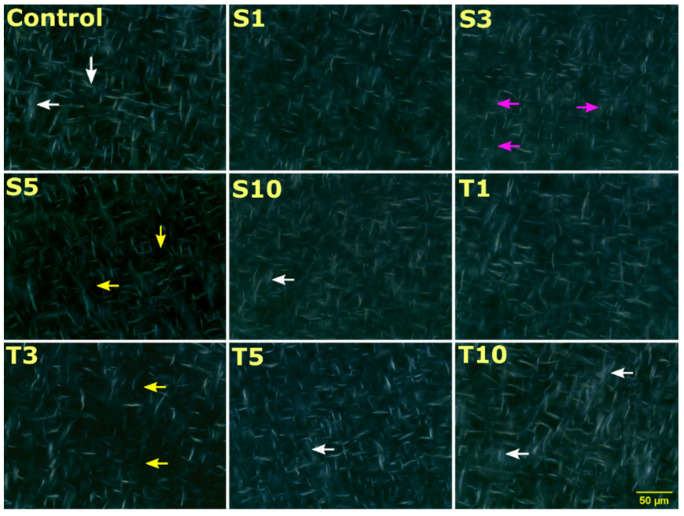
Polarized light micrographs of oleogel formulations. The details of abbreviations used are provided in Section 4.2 (Table 5).

The polarized light micrographs can be regarded as a better way to visualize the crystalline fat structures. The formed crystals appear bright, and the amorphous region appears as a black background. The obtained micrographs from polarized light microscopy further confirmed a mesh-like frame made up of needle/fibre morphology in all the formulations (Figure 5). The formation of the mesh or needle network can occur through the entanglement of the fat crystals. This morphology results from arrangements made by n-alkanes and wax esters that form a needle network in the structured oil [37]. The van der Waal interaction between the hydrocarbon chain and ester is responsible for the molecular organization and lateral crystal growth to form needle morphology in the oleogels [36]. Specifically, monoglycerides present in SFW can be accountable for crystallizing the fatty acid chain through supercooling [38]. The existence of differences in melting and crystallization temperatures is referred to as the supercooling effect. Some researchers have reported the needle-like structure to be an artifact of platelet morphology [39]. In some formulations, it seems that the fat crystals appeared as a bundle of several fibres (white arrow). In these structures, the needles were arranged together in several sized arrays that created bright visible zones. The needle length is distinctly long in the control sample, which appears to exist on the top of solid architecture. This information was not evident through the bright field images. The possible explanation for such an appearance can be the crystalline region made up of many fibres. On adding SPAN-80, the crystalline regions were dominantly observed in S1, S3, and S10, except for S5, where the amorphous area (or less crystalline area) is distinctly visible (yellow arrow). This can be the role of critical concentration in S5. Another observation in S3 was made for the presence of large globular shaped amorphous regions (pink arrow), which was not majorly seen in any formulation. Similarly, the addition of TWEEN-80 expressed a few amorphous regions in T3, which were rare in T1, T5, and T10, where the crystalline areas were dominant and spread throughout the matrix.

### 2.4. Mechanical Study

#### 2.4.1. Stress Relaxation Study

**Figure 6:**
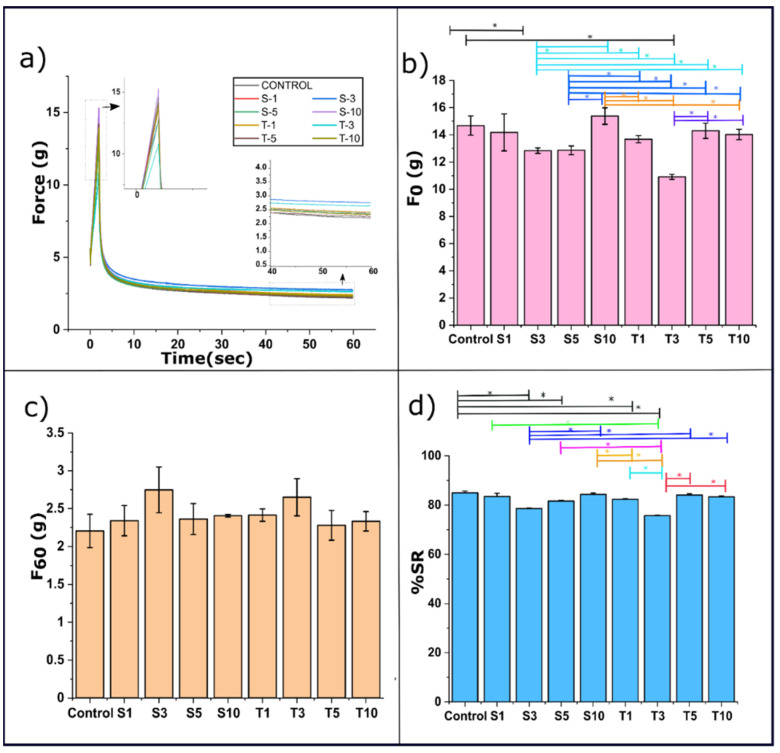
Mechanical properties of the oleogels. (**a**) Stress relaxation profile, (**b**) F_0_ values, (**c**) Residual elastic force in oleogels, and (**d**) %SR values. The values in the graph are denoted as the mean of the triplicate ± standard deviation (*p* < 0.05). The significantly different values are marked with *. The details of abbreviations used are provided in Section 4.2 (Table 5).

The viscoelastic properties of the prepared oleogel were studied using the stress relaxation (SR) profiles (Figure 6a). Once the oleogel is externally deformed, the wax network and the fluid pressure of the entrapped oil together exert the stress on the probe, which is displayed in the SR profile [40]. The firmness of the formulation is predicted using the maximum attained force (F_0_). Since the strained condition was maintained for 60 s, after achieving the maximum force, there occurs a decrease in the force values with an increase in time. On inclusion of SPAN-80, the F_0_ values of S1 showed similarity to the control. However, a further rise in emulsifier content in S3 caused a marked reduction of the F_0_ value of S1. The fragile nature of S3 may explain this observation. A further increase of SPAN-80 did not affect the firmness of oleogels and appeared similar to the control. Among the SPAN-80 formulations, S3, S5, and S10 displayed equal firmness to S1. Additionally, the F_0_ values of S3 and S5 were also identical to one another. However, the increased firmness in S10 was statistically significant from both S3 and S5. The increased firmness in S10 can be due to the increased linkage points within the fibrous network. These linkage points could be modulated by the SPAN-80 molecules [41].

Similar to the SPAN-80 formulation, only T3 among TWEEN-80 formulations showed a significant reduction in the F_0_ value from the control. The rest all the formulations hold comparable firmness to control. Among TWEEN-80 formulations T5 and T10 have similar firmness to that of the control. However, the decrease in firmness value of T3 from both T5 and T10 was notable, wherein T5 and T10 had identical firmness.

It was observed that the reduction in the F_0_ values of S3 and T3 was significant compared to the control. This observation advocates that adding 3 mg of SPAN-80 and TWEEN-80 in the said oleogel acts as a critical concentration point where the oleogels become more fragile. The polarized light micrographs can explain the reduced firmness, which displayed more amorphous regions (Figure 5) in these formulations. The micrographs can expound the poor mechanical strength in the said formulations [42]. A denser crystalline network of oleogelator provides better mechanical strength and improves the ability to hold the oil phase. A lower firmness of S3 and T3 oleogels also suggests their better ability to spread [43]. A detailed comparison of F_0_ values in all the prepared oleogels is represented in Figure 6b.

The force value at the end of the relaxation profile depicts the residual elastic force (F_60_), which is described in Figure 6c. This force decay occurs by the significant rearrangements in the gelator molecules, disturbance in network structure, and the break in the fibre network [44]. No significant difference was observed in the F_60_ values among the formulations. The possible insignificant rise of F_60_ value from control in both types of formulations is justified by the ring structure at the carbonyl group in sorbitan, which offers rigidity to the 3-dimensional network of fat [30]. Further, the %SR was calculated using the values of F_0_ and F_60_ (Equation (7)). The %SR value represents a sample’s ability to absorb the energy during a strained condition. The values of %SR follow a different trend in SPAN-80 and TWEEN-80 formulation. The inclusion of SPAN-80 showed no effect on %SR of S1 from the control. However, a significant decrease from the control was observed in the %SR of S3 and S5. S3 showed the lowest %SR value among the SPAN-80 formulation. A lower %SR of the SPAN-80 containing oleogels suggests an increased rigidity to that of the control. Again, the %SR in S10 was observed to be similar to that of the control. Among the SPAN-80 formulations, not many alterations were observed in the %SR values except for a noteworthy increase from S3 to S10. Interestingly, T1 and T3 showed a significant decrease of %SR from control. However, SR behaviour was similar to the control at the higher TWEEN-80 content, i.e., T5 and T10. A trend of T5 ≈ T10 ≈ T1 > T3 was observed for %SR of TWEEN-80 formulations. The inclusion of both emulsifiers at lower content may reduce the reorganizational capability of gelator molecules. This significant decrease also means a high elastic component, as confirmed through F_R_ values and low viscous components of the oleogels [45]. It is quite evident that even a lower amount of emulsifier affects the mechanical properties of the formulation [41]. The possible reason behind this can be the alterations observed through the micrographs in the size, morphology, and number of fat crystals.

Further modelling of the SR profile of oleogels was done using Weichert’s model. The model includes many Maxwell units (spring and dashpot) connected in parallel [46]. Weichert’s model considers relaxation to occur in consequent series. Evaluating the model’s usefulness, in our study, we have used this model to analyse the viscoelastic property of the formed oleogels with two characteristic times represented in Equation (1). The correlation coefficient (R^2^) value among the experimental and model data in all the oleogels formulation was >0.99.

(1) $$P\;={\;P}_{0}\;+{\;P}_{1}e^{\left(-\frac{t}{\tau 1}\right)}+{\;P}_{2}e^{\left(-\frac{t}{\tau 2}\right)},$$

where P, P_0_, P_1_, and P_2_ are spring elements, and τ_1_ and τ_2_ are time constants of dashpots.

The P_0_ value helps interpret residual force, mechanical stability, and inherent elastic properties of the oleogel at the end of the stress relaxation process (Table 1) [44]. In SPAN-80 formulations, the P_0_ value of all formulations appeared similar to the control. However, the rise of P_0_ in S5 from S1 and S3 was found significant. The addition of TWEEN-80 caused a substantial and subsequent increment in the P_0_ value from control till T3. The increment of P_0_ from T1 to T3 was found to be significant. A further rise in the emulsifier content did not affect the P_0_ value. The P_0_ value of T3 was the highest among all the formulations, which suggests a better inherent mechanical property in this case (Table 2). The time constants, τ_1_ and τ_2_, provide information regarding instantaneous relaxation time and delayed relaxation time. When stress is applied to the gel, the immediate relaxation time corresponds to the molecular rearrangement of the constituents of stressed oleogels. On adding SPAN-80 to oleogels, the value of τ_1_ was significantly reduced from the control in S1 and S3. A further rise in emulsifier content in S5 caused a significant increase in the τ_1_ from control, S1, and S3. This observation suggests slower molecular rearrangement inside S5 oleogels when the force is applied. The possible reason for this is the heterogeneous networking, as shown in polarized light micrographs (Figure 5). At the maximum SPAN-80 content in S10, there was no significant difference in the value of τ_1_ from control and other formulations. On adding TWEEN-80 to the formulations, the value of τ_1_ was significantly reduced in T1 and T3, from the control, although τ_1_ in T1 and T3 appeared similar. In T5 and T10, a considerably higher value of τ_1_ from control was observed. The rise in τ_1_ of T10 from T5 was found to be significant. The delayed relaxation time scripts the disruption in the oleogel network if the stress is maintained for a longer time [47]. On adding SPAN-80 to oleogels, the value of τ_2_ was significantly reduced from the control in S1. Further addition in the emulsifier content in S3 displayed a similar τ_2_ value to that of the control and S1. A significant reduction in the τ_2_ value from the control and S3 was observed in S5. At the highest emulsifier content, the τ_2_ value of S10 appeared similar to the control and other SPAN-80 formulations. However, among TWEEN-80, all the formulations showed a significant reduction from control in the τ_2_ values. The longer relaxation time on the addition of TWEEN-80 to oleogels indicates the lesser likelihood of oleogels undergoing network architecture breakage [45]. In other words, the gelator network formed by the TWEEN-80 oleogels can maintain their structural integrity for an extended time under stressful conditions.

#### 2.4.2. Spreadability Analysis

**Figure 7:**
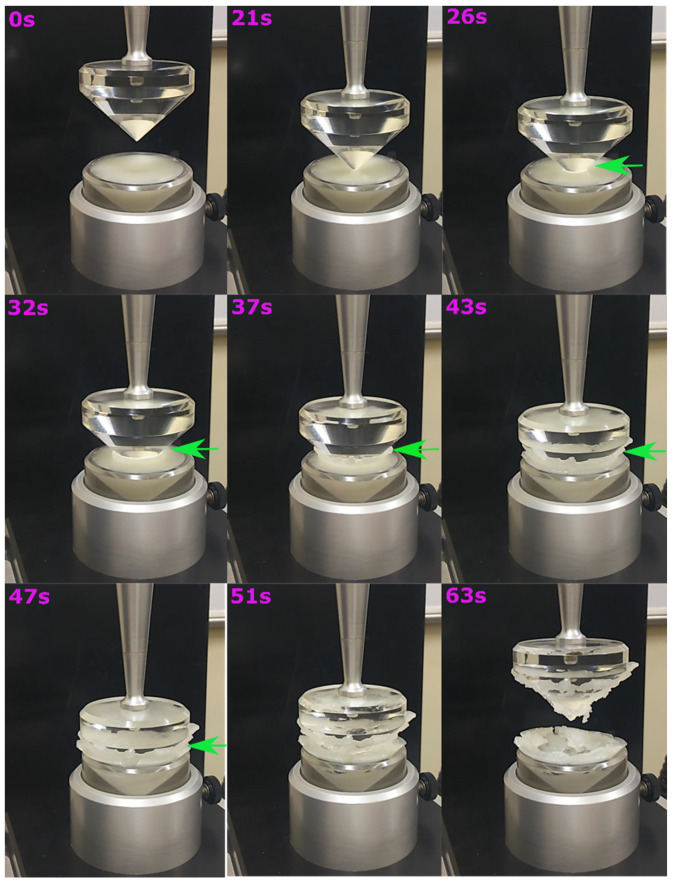
Time-based stages of the spreadability cycle of oleogel indicating its semi-solid nature. The green arrow indicates the increased diameter of the annulus formed by the male cone.

Figure 7 represents the stages of the spreadability cycle. These stages help in the visualization of the semi-solid nature of the formulated oleogels. A trigger force of 5 g was set for the analysis. Afterward, the male cone penetrated oleogels at a rate of 0.5 mm/s to a depth of 13 mm. At 26 s, after the penetration of the male cone, a clear annulus is visible (green arrow), whose diameter slowly decreases with increasing depth. The male cone compresses and extrudes the oleogel from the female cone (Figure 7, 43 s), spreading evenly between the male and female cones. Figure S1 represents the spreadability profiles of the formulations. The peak value in the positive curve represents the firmness (F_0_), and the area under the positive curve depicts the amount of energy (C_0_) required to deform the oleogels. The probe, while returning, incorporates the information of cohesiveness and resistance of the oleogels to separate from the cone. The peak value in the negative curve denotes adhesive force/stickiness (S_0_). The area under the negative peak is the measure of adhesiveness (A_0_). Unlike the SR study, we did not observe any differences in the spreadability properties as the parameters (Table S3) discussed above appeared similar in all the formulations. This observation suggests that the bulk properties of oleogels remained the same even after the addition of the emulsifiers.

### 2.5. Molecular Analysis

#### 2.5.1. FTIR Spectroscopy

FTIR spectroscopy was used to estimate the chemical nature of raw components (i.e., SO, SFW, SPAN-80, and TWEEN-80) used to prepare the oleogels (Figure S1). The spectra observed in SFO and SFW resemble triglycerides, which are crucial components in edible oils [48]. The characteristic spectra of SO included a band at 3007 cm^−1^ due to the C–H stretching vibration in =C–H (*cis*). Since SO is composed of linoleic and oleic acids consisting of a significant amount of unsaturated fats, they are the possible explanation behind the appearance of this peak. The bands representing the C–H stretching vibration in methylene and methyl groups are present at 2924 cm^−1^ and 2852 cm^−1^, respectively [48]. The peaks further confirmed the presence of these two groups in spectra of SO at 1459 cm^−1^ and 1377 cm^−1^, which can be ascribed to the C–H bending vibration in methylene and methyl groups [48]. A comparatively larger peak at 1742 cm^−1^ was observed due to the –C=O double bond stretching vibration from the ester groups. These groups are present in abundance in SFO. The observed bands at 1236 cm^−1^, 1159 cm^−1,^ and 1097 cm^−1^ represent a –C–O stretching vibration corresponding to the ester group [49]. Further, the bending vibration of trans –CH=CH– is noted at 967 cm^−1^ and the rocking vibration of –(CH_2_)_n_ at 722 cm^−1^ [49]. The FTIR spectra of SFW showed asymmetric stretching vibration of methyl group through a band positioned at 2954 cm^−1^. The band located at 1375 cm^−1^ corresponds to the symmetrical bending vibration of CH_3_ [50]_._ Further, a distinct peak at 2916 cm^−1^ and 2846 cm^−1^ resembles the symmetric axial deformation of CH_2_. The spectral band at 1471 cm^−1^ and 1463 cm^−1^ stands for symmetric angular stretching or bending vibration of CH_2_ [50]. Two continuous bands at 728 cm^−1^ and 718 cm^−1^ correspond to the in-plane CH_2_ deformation [51]. A distinct peak at 1732 cm^−1^ corresponds to the stretching vibration of –C=O present in ester.

Structurally, SPAN-80 and TWEEN-80 consist of hydrophilic groups such as sorbitan and polyethylene glycol, which comprise several polar groups. The core lipophilic groups in them are long hydrocarbon chains of fatty acids, fatty alcohols, and esters. The band at 2922 cm^−1^, and 2852 cm^−1^ in SPAN-80 and 2926 cm^−1^, and 2863 cm^−1^ in TWEEN-80 spectra corresponds to the C–H stretching vibrations. However, these two bands occur as distinct peaks in the case of SPAN-80 but merge for TWEEN-80. The possible reason can be less branching in the structure of SPAN-80 as compared to TWEEN-80 [52]. Additionally, peaks in the range 720–946 cm^−1^ implicate the vibration of C–H deformation in the two emulsifiers [53]. The spectral band at 1738 cm^−1^ of SPAN-80 and 1734 cm^−1^ of TWEEN-80 is for stretching vibration of C=O of ester groups present in these emulsifiers. The peak 1463 cm^−1^ and 1377 cm^−1^ along with 1457 cm^−1^ and 1350 cm^−1^ corresponds to C–H scissoring vibration in SPAN-80 and TWEEN-80, correspondingly [54]. Stretching vibration of C–O–C present in the ester group was depicted by the peak present at 1171 cm^−1^ in SPAN-80. However, in TWEEN-80, the C–O–C stretching vibration was identified with the band at 1095 cm^−1^.

**Figure 8:**
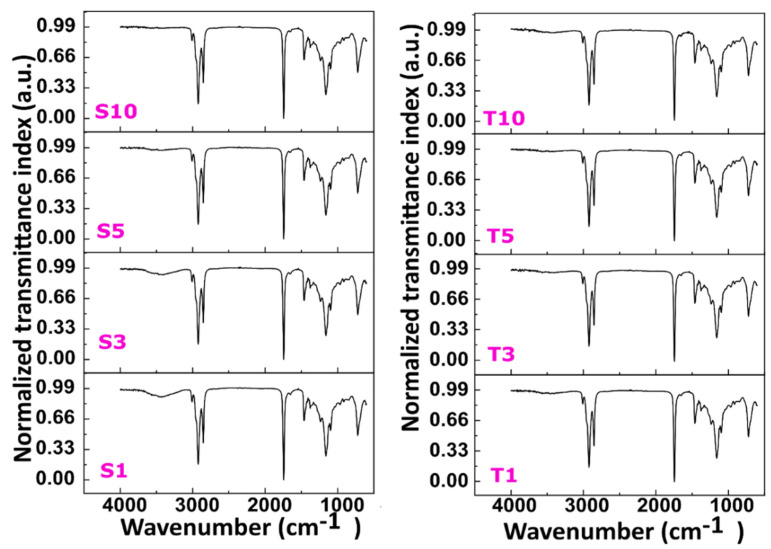
FTIR spectra of oleogels. The details of abbreviations used are provided in Section 4.2 (Table 5).

FTIR spectroscopy was used to obtain information regarding the interactions among the components in the control oleogels (Figure S2). We have noticed that all our formulations (Figure 8) showed an overall IR spectra pattern similar to that of the control. The recorded IR spectra showed single peaks at 722 cm^−1^ and 1461 cm^−1^. This accounts for CH_2_ rocking vibration and CH_2_ and CH_3_ bending, respectively. FTIR spectra are capable of providing information regarding the acyl chain packaging in the lipid systems. The group splitting of CH_2_ rocking (722 cm^−1^) and CH_2_ bending (1461 cm^−1^) mentioned before is common to orthorhombic subcell [55]. This splitting is specific for orthorhombic packing and is absent in subcell hexagonal and triclinic packing [56]. Another set of significant peaks occurred at 2852 cm^−1^ and 2922 cm^−1^, resulting from symmetric and antisymmetric CH_2_ stretching [57]. A distinct peak at 1163 cm^−1^ represents symmetric—stretching. The prominent peak at 1742 cm^−1^ corresponds to the C=O aliphatic ester groups of triglycerides present in SFO and SFW [58]. A minor peak at 3010 cm^−1^ was recorded in all the oleogel formulations, also present in SFO. This peak is contributed to the bond vibration of the alkene (=C–H) from SFO. The presence of this band in the IR spectra indicates a high degree of unsaturation. There was no marked fluctuation in the peaks mentioned above in the IR spectra of SPAN-80 and TWEEN-80 formulations, possibly due to the meagre amounts of emulsifiers. The absence of any band shift was confirmed by taking the instrument’s spectral resolution (4 cm^−1^) into consideration. Since no change in the peak position was observed, the obtained FTIR results confirm no significant difference in the chemical interaction in the emulsifier-containing oleogels compared to the pristine oleogel. The gel formation is solely based on non-covalent interactions such as hydrogen bonding and van der Waals attraction.

#### 2.5.2. XRD Diffraction Patterns

**Figure 9:**
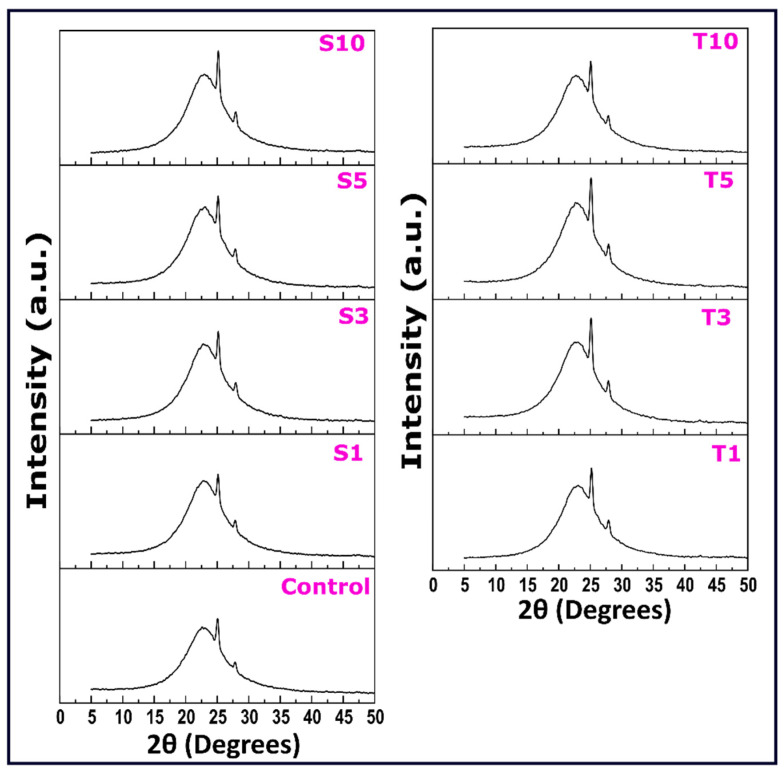
XRD diffractograms of oleogel formulations. The details of abbreviations used are provided in Section 4.2 (Table 5).

The polymorphism of the oleogels was studied through the XRD diffractograms. The diffraction patterns of oleogels prepared by adding a varied amount of emulsifiers are shown in Figure 9. The lateral packing of the fatty acid chains in the 3-dimensional networks is obtained by the short d-spacings, which can be calculated from the diffractograms at wide angles. The lateral packing of triacylglycerol has been reported through three classic organizations. These organizations include the least stable α form, metastable β′ form, and most stable β polymorphs [59]. These polymorphs exist in subcell packing as hexagonal, orthorhombic perpendicular, and triclinic parallel [60]. Further, palmitic acid in sunflower oil promotes the β′ polymorph in the oleogels [61]. As previously mentioned, wax comprises various molecules, i.e., hydrocarbons, esters, fatty alcohols, and fatty acids. The diffraction pattern of control showed a broad peak at 22.6° 2θ, corresponding to Bragg’s distance (d-spacing; interplanar spacing) of 4.56 Å (Equation (1)). A sharp peak was observed at 25.06° 2θ with a d-spacing of 4.12 Å. The third sharp peak, positioned at 27.78° 2θ, has an intensity lower than the previous one and a d-spacing of 3.72 Å. The three mentioned peaks are called “short spacing peaks,” associated with hydrocarbon chains’ lateral packing. The d-spacing value of 4.12 Å and 3.72 Å is an indication of the presence of β′ polymorph of triacylglycerols [62]. These d-spacing values correspond to the orthorhombic subcell packing [36]. The β′ polymorph of fats in food products such as margarine and shortenings is responsible for the even texture, spreadability, and mouthfeel.

Incorporating the emulsifiers increased the intensity of these peaks, which gives an idea that the emulsifiers, at the content used, have assisted in the lateral packing of chain in the fat network. Inclusion of SPAN-80 has displayed these peaks roughly at 22.94° 2θ, 25.145° 2θ, and 27.92° 2θ. The interplanar spacing displayed the following values respective to the mentioned peaks, i.e., 4.49 Å, 4.10 Å, and 3.707 Å. Similarly, in TWEEN-80 oleogels, the broad peaks occur at ~22.83° 2θ, having an interplanar spacing of 4.51 Å. The other two peaks were positioned at 25.12° 2θ and 27.92° 2θ with the corresponding interplanar spacings of 4.11 Å and 3.71 Å. These peak positions are related to the presence of β′ polymorphs of wax crystals whose stability is somewhere between polymorphs α and β. Additionally, the β′ form of wax crystal displays needle and fine grain-like microstructures, which was evident in micrographs from our study [63]. A better understanding of d-spacing and their trend with the usage of emulsifiers is discussed in the next section. The observed shifts in the peak position of oleogels added with emulsifier compared to control can be due to the defect caused in the fat network.

The profiles were deconvoluted in Origin Pro software using the Gauss peak fitting function to understand the crystal properties better. The obtained deconvoluted data was capable of displaying five characteristic peaks in all the formulations. Further, this data helped to calculate the crystallite size (D), lattice strain, and dislocation density (Table 2). On adding SPAN-80 in oleogels, the average d-spacing values showed an increment in S3, and the rest of the formulations showed values similar to control. However, the addition of TWEEN-80 did not show any significant change in the average d-spacing values. The addition of SPAN-80 and TWEEN-80 to the oleogels has improvised the crystallite size compared to control. Additionally, the large crystal size is responsible for high crystallinity, allowing wax molecules to form stable polymorphs [64]. This suggests the possibility of liquid emulsifiers used in our study to act as crystal modifiers. A deep analysis of the parameters clear that although the S3 micrographs have displayed more amorphous regions, the crystallite size in them is most significant compared to the control and other SPAN-80 formulations. The large crystallite size may be due to the slow crystallization rate of the fat molecules. The inclusion of 3 mg of SPAN-80 in the oleogel supported the fat crystal growth. Similarly, the reduced value of lattice strain and dislocation density backs fewer crystal defects in S3, which promoted the formation of the larger fat crystals. Among the TWEEN-80 formulation, T3 has shown the largest crystallite size and lower lattice strain and dislocation density. Previous work has demonstrated the potential of polysorbates to co-crystalize with fats, thus improving crystal growth [27]. In a nutshell, it can be inferred that the inclusion of both the liquid emulsifiers has introduced fluctuations in the structural architecture of the wax network. However, formulations S3 and T3 have been found to support fat crystal growth.

### 2.6. Thermal Analysis

#### 2.6.1. Gelation Kinetics

**Figure 10:**
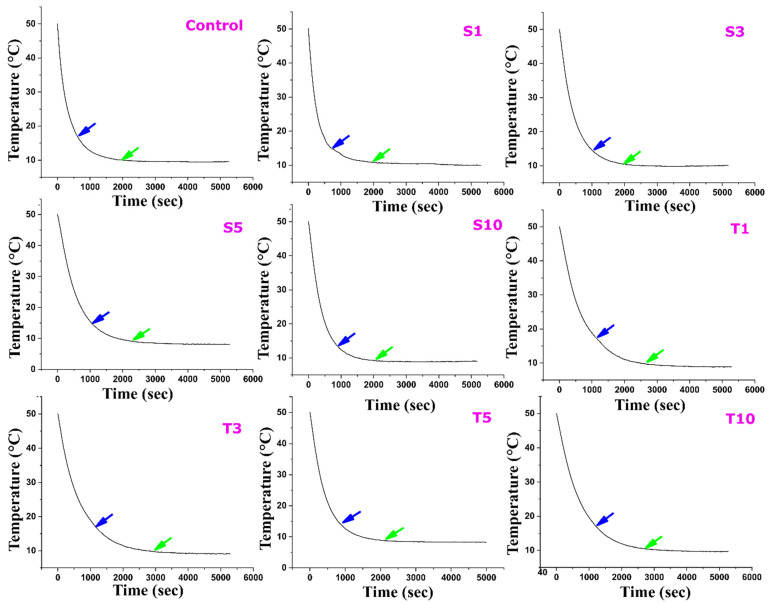
Gelation kinetics of all the formulations. (Blue arrow: onset of secondary crystallization (s), Green arrow: time to reach thermal equilibrium (s). The details of abbreviations used are provided in Section 4.2 (Table 5).

Gelation/crystallization is a process of arranging the triacylglycerols into a compact structure. This arrangement is possible due to the physical and chemical bonds between the triacylglycerol, thus restricting its movement [65]. The gelation behaviour of fats and its understanding is essential to safeguard required industrial applications of fat-based products. The conventional mechanism of lipid structuring consists of three stages, i.e., nucleation, crystal growth, and maturation. These three stages assist in the formation of the fat crystal lattice. The graph of gelation kinetics (Figure 10) depicts the three stages of fat crystallization. The stages include initial, intermediate, and final saturation phases [57]. The initial step is marked by a sharp decline in the temperature of formulations and is marked in blue arrows in the representative temperature versus time graphs. This can be correlated to the nucleation phase of lipid structuring and is a sign of secondary crystallization. The secondary crystallization of fats in the control is marked at a time point of 621 s, and this point in the graph differs among all the formulations. An essential aspect of our study is understanding the impact of the chosen liquid emulsifiers on fat crystallization. The effect of emulsifiers on the gelation kinetics can be due to the different organization of crystals in the arrangement or by forming imperfections. Emulsifiers with dissimilar hydrophobic properties cause substantial effects on the crystallization of fats [66]. This is usually done by altering the kinetics of crystallization or by varying transitions in the polymorphs. The addition of SPAN-80 in the oleogel lengthened the onset of secondary crystallization in the case of S1, S3, and S5 upon increasing the emulsifier’s amount (Table 3). However, S10 showed a rapid onset (908 s) of secondary crystallization in the oleogels. This agrees with the previously reported data where a higher proportion of lipophilic emulsifiers have displayed reduced onset of crystallization induction time [67]. In TWEEN-80 formulations, the onset of secondary crystallization was slow in T1, T3, and T10 and was comparatively rapid in the case of T5.

Beyond the blue arrow, a continuous transformation is visualized in the crystallization process of all the formulations. Finally, the green arrow scripts the accomplishment of thermal equilibrium in the crystallization kinetics curve, which, like the onset point, differs among the formulations. In our study, control and S1 have reached equilibrium at the earliest ~1900 s. Further, an increase in the amount of SPAN-80 delayed the attainment time of thermal equilibrium till S5. Since S10 showed a rapid initiation of secondary crystallization, it took less time than other formulations to reach thermal equilibrium (~2092 s). A similar trend in the initiation stage and thermal equilibrium was observed in TWEEN-80 formulations. Most of the TWEEN-80 oleogels showed delayed onset of secondary crystallization and attainment of thermal equilibrium between the two formulations. The bulky hydrophilic head and kinked carbon chain of TWEEN-80 are possible reasons that can affect the process of fat crystallization [27].

The crystallization curve in the early portion (0–200 s) was then analysed in-depth. The initial portion of the curve was then fitted to an exponential decay function, which is represented as;

(2) $$y\;={\;\mathrm{ae}}^{-\mathrm{kt}}$$

where ‘a’ is the initial temperature (°C), ‘k’ is the crystallization rate, and ‘t’ is time (s).

As a general observation, the inclusion of both the emulsifiers reduced the crystallization rate compared to the control. This suggests the possibility of alteration in the kinetics of gelation. This observation can be reasoned to the presence of sorbitan esters. The sorbitan esters not only slow down or inhibit the polymorphic transitions and stabilize the β′ polymorphs of triacylglycerols [15]. A low crystallization rate of S3 further supports the fewer crystal defects, as confirmed from XRD. Similarly, an even lower value of rate in S5 can be the reason for the delay (~2300 s) in thermal equilibrium. Again, the addition of TWEEN-80 showed reduced crystallization rates to a greater extent in all the formulations, notably in T3 and T5. The possible reason can be the observed larger crystallite size in these formulations. In addition, the giant hydrophilic head and the unsaturated carbon chain of TWEEN-80 can cause a hindrance and thus delay the crystallization rate [27]. A research group has also attributed the delayed crystallization to the interference of emulsifiers in the packing of crystals [27]. The reduction in the rate values of crystallization is a combined effect of nucleation rate, crystal type, morphology, size, crystal arrangement, and growth [68].

#### 2.6.2. DSC Analysis

**Figure 11:**
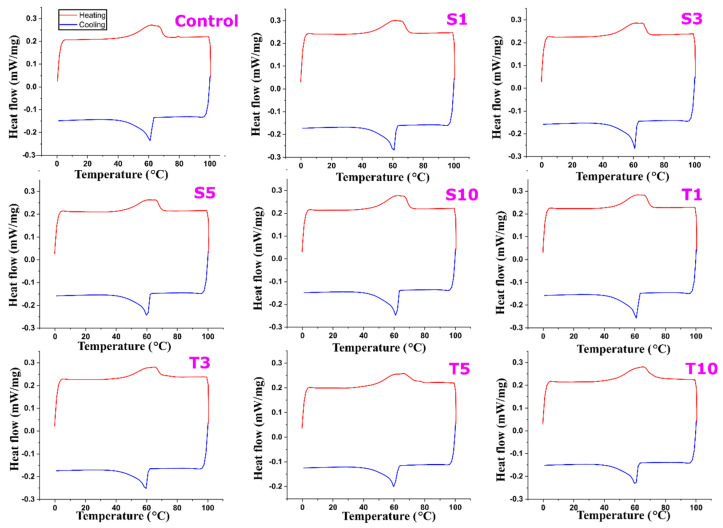
DSC thermograms of formulated oleogels. The details of abbreviations used are provided in Section 4.2 (Table 5).

Plant waxes are of natural origin, which attributes to their compositional differences and physical behaviour. The melting and crystallization behaviour is one of the physical parameters that were observed for the prepared oleogels. The colligative properties of wax help them by solubilizing into the vegetable oil. This results in the decrement of melting and crystallization temperature of wax oleogels. These temperatures of wax-based oleogels are usually lower in comparison to neat waxes [36]. Any change in the physical state of oleogel or polymorphic transitions is associated with the difference in the absorption (endothermic) or release (exothermic) of heat. This heat flow can be measured through DSC as a function of time and temperature. Figure 11 represents the DSC heating and cooling profiles of all the formulations. The obtained heating profiles of all the formulations displayed a broad peak and one shoulder peak in the temperature range 44–70 °C. The melting onset in all our formulations is from ~40 °C. Deconvolution of the broad peak using Origin Pro software was performed to reveal the positions and area of the peaks. It was found out that the broad peak comprised of two endothermic signals (Table S3) in the temperature range 60–70 °C. When present in different polymorphic forms, fats usually appear as more than one endotherm [69]. The control sample’s melting temperature/peak temperature (T_m_) was observed through a significant peak at ~62 °C along with a shoulder peak at 65 °C. Previous work reported a T_m_ of 58.4 °C for oleogel containing 3% SFW along with the possibility of increased values at higher wax concentration [70]. The peak melting temperature of SFW and olive oil oleogels has been reported to be between 58 and 63 °C in literature [71]. The range of observed melting points in our study corresponds with that of triglycerides melting behaviour [72]. β form, the most stable form of fats, has the highest melting point [73]. SFW is a high melting wax due to the presence of highly saturated or long-chain fatty acids. Since our XRD diffractograms confirmed the presence of β′ polymorph, there is a possibility of melt-mediated transformation in the formulations. This transformation suggests the appearance of a stable (β) fat polymorph when a metastable (β′) form is heated over its melting point [73]. However, incorporation of a emulsifier at a certain amount slightly changed the peak positions. On adding SPAN-80 to oleogels, the melting temperature reduced compared to the control in all t formulations except for S3, where T_m_ was observed at 64 °C. This is interesting because a high melting temperature suggests better formulation stability and correlates with slower gelation kinetics and fewer crystal defects in the S3 discussed above. Further, the conversion of β′ to β is associated with the formation of a crystal with a large size [74]. The presence of a larger crystallite size displayed through XRD analysis in S3 further supports its better stability among SPAN-80 formulations. However, the addition of TWEEN-80 has increased the T_m_ value in the case of T3 and T10. The result is yet again in line with the results obtained from the XRD analysis. Crystallization onset and peak temperature have also been tabulated (Table S4). The crystallization of waxes in liquid oil starts with its supersaturation in the oil and, thus, can be affected by the amount of wax used as a gelator [75]. For every formulation, the peak in the cooling profile occurs at ~60 °C irrespective of the addition of a liquid emulsifier. The appearance of a similar peak indicates the presence of similar polymorphs i.e., β form in all the formulations.

### 2.7. Drug Release Analysis

**Figure 12:**
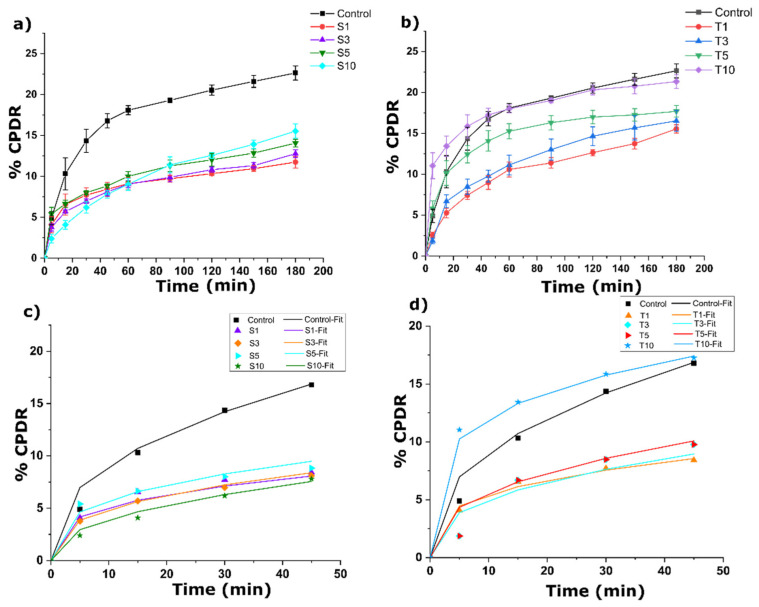
Drug release profile (**a**) SPAN-80 formulations, (**b**) TWEEN-80 formulations (**c**) PS model fitting for SPAN-80 formulation (**d**) PS model fitting for TWEEN-80 formulations. The details of abbreviations used are provided in Section 4.2 (Table 5).

Curcumin is a lipophilic compound with enormous health benefits as a herbal medicine [76]. It is crucial to develop curcumin formulations that have improved bioavailability. The bioavailability at the site of action will depend on the release of curcumin from the oleogels. This release was calculated as cumulative percentage drug release (CPDR). At the end of the experiment (after 180 min), the emulsifier-containing oleogels showed a marked reduction in the %CPDR compared to the control (Figure 12). The inclusion of hydrophobic surfactants in the oleogel might have increased the lipophilicity of the wax oleogels. Among the SPAN-80 formulations, S1 and S3 showed similar %CPDR. S5 showed a substantial increase in the %CPDR from S1 and S3. At the highest SPAN-80 content, the release was found to be identical to S5; however, it was greater than S1 and S3. The addition of TWEEN-80 in T1 and T3 showed similarity in the drug release after 180 min. The formulation T5 led to a similar %CPDR to T3, though this value was higher than T1. At the highest TWEEN-80 content, there was a significant rise in the %CPDR from T1, T3, and T5.

A further understanding of the drug release mechanism was developed using Peppas Sahlin (PS) model fitting with correlation coefficient >0.99 (Table 4), which is represented in Equation (2) [77]. The obtained correlation coefficient was an indication of the good fit of the experimental data with the modelled data. The PS model combines the effect of Fickian diffusional release and relaxation release of drugs from the polymers. The PS model was initially developed to evaluate the drug release from the hydrophilic matrix; however, attainment of good fit in this study suggested its implementation to hydrophobic polymers [78].

(3) $$F\;={\;K}_{d}t^{m}+{\;K}_{r}t^{2m}$$

where F indicates the portion of the solute released; K_d_ and K_r_ are constants related to Fickian kinetics and relaxation kinetics, respectively; m is the Fickian diffusional exponent, which prevents the controlled release of the drug, and t is the time at which sampling took place.

The Fickian diffusion constant (K_d_) for every SPAN-80 formulation was significantly reduced in comparison to the control. The K_d_ value subsequently decreased till S3, however, this value showed a rise in S5 from S1 and S3. An increase of SPAN-80 content in S10 showed the least K_d_ among all the formulations. Slightly higher K_d_ values of S1 and S5 among SPAN-80 formulations are attributed to the void spaces observed in these formulations’ micrographs. The voids may have aided in a comparatively faster release of curcumin. The addition of TWEEN-80 to the formulations displayed a subsequent decrease in the K_d_ value till T3. Formulation T3 had the lowest K_d_ value among all the formulations, suggesting an equal contribution of diffusion and relaxation in the release process. Further rise in the emulsifier content in T5 displayed a significantly higher K_d_ value than T3, but this value was found to be similar to the control. Formulation T10 showed the highest K_d_ value, which was even higher than the control, suggesting a dominance of diffusional mechanism in this case. The higher values of K_d_ than K_r_ (or K_d_/K_r_ > 1) in all the formulations indicated the importance of diffusional contribution towards the curcumin release from the SFW-based oleogels [79].

## 3. Conclusions

A continuous change in the global scenario of dietary patterns and guidelines has prompted scientists to discover the alternative of trans fats. An opposite substitute of trans-fats should not compromise the functional integration of the food products, which otherwise will not be accepted by the consumers. The development of oleogels in lipid research has emerged with an exciting potential to replace saturated fats. The present study showed the potential of SFW to bind the SFO, which was formulated using the heating-and-cooling method. Further, the effects of liquid hydrophilic and hydrophobic emulsifiers were studied through various techniques such as colorimetry, microscopy, texture analyser, FTIR spectroscopy, XRD, crystallization, and DSC. Irrespective of the emulsifier treatments, all the 5% SFW oleogels have shown more than 99% OBC. The formulated oleogels had an ivory appearance and smooth touch, ideal for many food applications, including shortenings and margarine [71]. Our study revealed an amount of 3 mg of both SPAN-80 and TWEEN-80 in 20 g of oleogels is optimum for structural, molecular, and thermal parameters when included in 5% SFW oleogels. The surface topography images displayed the uniform distribution of globular structures. The addition of both the emulsifiers has shown an alteration in globular size and distribution. Topographs of S3 showed crystals that were most noticeable and bright. Comparable globular structures were also observed in T3. The wax esters of SFW were able to form a fibrous network of varied length, thickness, and branching, which were clearly visible under bright field micrographs. The SPAN-80 formulations, notably S3, were more hyperbranched and dense than TWEEN-80 formulations, which specify a good mechanical strength and oil binding capacity. The examined viscoelastic properties of the oleogels through SR profiles revealed alteration in the firmness, residual elastic force, and %SR. These properties were not entirely dependent on the emulsifier’s content. The formulations, S3 and T3, displayed lower spreadability and higher inherent elastic properties. All the formulations showed an overall IR spectra pattern similar to that of the control, thus suggesting a not significant difference in molecular aspects. The XRD diffractograms revealed the presence of metastable β′ form in all the formulations. Further, S3 and T3 showed large crystallite sizes compared to the control sample, suggesting that the emulsifiers were able to induce optimum conditions that supported the crystal growth of the fats. The crystallization kinetics study under isothermal conditions showed that both the emulsifiers had reduced the crystallization rate in all the formulations as compared to the control sample. A slower crystallization rate in S3 and T3 supported the formation of larger crystal sizes with the possibility of fewer crystal defects. An interesting case of melt-mediated transition of β′ to β was revealed through the heating profile of DSC thermograms. This was prominently observed in S3 and T3, further suggesting the presence of a stable polymorph in these formulations. Based on the observation mentioned above, it can be said that the minimum acceptable amount of emulsifiers in food products still have the potential to act as crystal modifiers. SFW oleogel with improvised properties in this way can act as a fat replacer in many bakeries and other food products.

## 4. Material and Methods

### 4.1. Materials

Commercial refined sunflower oil (Fortune sunlight, Kutch, Gujarat, India), used for the oleogel preparation, was purchased from the local supermarket. Sunflower wax pellet was procured from Vijay Impex, Chennimalai, Tamil Nadu, India. SPAN-80 and TWEEN-80 were purchased from Loba Chemie Pvt. Ltd., Mumbai, India, and Himedia Laboratories Pvt Ltd., Mumbai, India, respectively.

### 4.2. Oleogel Preparations

Five percent of SFW was found to be the critical gelation concentration (CGC) for SFO. Hence, an oleogel of SFO containing 5% (*w*/*w*) of SFW was prepared, which served as the control formulation. In gist, accurately weighed SO and SFW were taken in a beaker placed in a water bath (80 °C) for ~20 min. The mixture was heated until all the wax was dissolved in the oil, and a homogeneous solution was formed. This clear solution was then kept at room temperature (25 °C) in the thermal cabinet for 30 min to encourage oleogel formation. Stock solutions of 0.1% (*w*/*w*) of SPAN-80 and TWEEN-80 were prepared in SO. A series of 1 mg, 3 mg, 5 mg, and 10 mg of SPAN-80 and TWEEN-80 added formulations were prepared following the composition as per Table 5. The rest of the process for the development of the formulations was the same.

### 4.3. Study of Oil Released and Oil Binding Capacity (OBC)

The study of oil released was performed initially to find out the OBC of formed oleogels. For this, an empty 2 mL previously weighed (a) Eppendorf was taken, and 1 mL of the molten sample was placed in the Eppendorf. The sample was then allowed to solidify, followed by weighing the Eppendorf (b). The Eppedorfs were kept at 4 °C for 24 h, after which they were centrifuged with Remi C-24 BL refrigerated centrifuge at 10,000 rpm (18 °C) for 15 min. The Eppendorfs were then inverted to drain the released oil. The remaining oil over the surface of the oleogels was removed using filter paper. The %oil released and OBC was calculated using Equations (3) and (4), respectively [78].

(4) $$\%\mathrm{Oil}\;\mathrm{released}\;=\;\{\frac{\left[\left(b\;-\;a\right)-\left(c\;-\;a\right)\right]}{\left(b\;-\;a\right)}]\;\times 100$$

(5) OBC=100−%Oil released

where, a is the 2 mL previously weighed Eppendorf; b is the solidified sample containing Eppendorf; and c is the final weight of Eppendorf after centrifugation and draining.

### 4.4. Colorimetric Analysis

Colorimetric analysis of the oleogels was done with a lab-developed colorimeter. The hardware of the colorimeter device consists of a light-emitting diode (LED) as a light source and Picam for imaging purposes [79]. The instrument was calibrated before the experiment with the standard white and black tiles. The analysis was done using oleogels that were placed in 35 mm Petri-dishes. Thereafter, the colour coordinates L*, a*, and b* values were measured.

### 4.5. Microscopic Analysis

#### 4.5.1. Surface Topography

The surface topography of the formulations was visualized using a Stereo Zoom Microscope (Model: SM-2TZ; Make: AMscope, Irvine, CA, USA). The microscope was equipped with an external eyepiece lens camera (AMscope MD500, Irvine, CA, USA). The samples for the study were prepared by transferring 5 g of the molten mixture in 35 mm petri-dish and solidifying the same as per the method reported in Section 2.1.

#### 4.5.2. Microstructure Visualization

The molten oleogels drop was placed on a glass slide followed by covering with a coverslip. The microstructure arrangement of the prepared oleogel was visualized using an upright bright-field compound microscope (Leica Microsystems, model: DM750, GmbH, Wetzlar, Germany), coupled with an in-house built polarizer. The micrographs were visualized both under bright-field and polarizing modes.

### 4.6. Mechanical Study

#### 4.6.1. Stress Relaxation

Mechanical properties of the oleogel were examined by stress relaxation (SR) studies. The study was conducted using the texture analyser HD plus instrument (Godalming, Surrey, UK). For the SR study, the oleogels were prepared in 100 mL polypropylene beakers. The initial trigger force was set as 5 g. The solidified oleogel was penetrated with a constant strain of 1 mm at a rate of 0.5 mm/s using an acrylic male conical probe (angle 45°). The strained condition was maintained for 60 s, and the values for change in forces were recorded. Then, the probe was brought back to its usual height, i.e., 30 mm from the surface. Using the force values, %SR was calculated for each oleogel sample using Equation (5) mentioned below:

(6) $$\%\mathrm{SR}\;=\frac{F_{0}-F_{R}}{F_{0}}\times 100$$

F_0_: Maximum force in SR curve

F_R_: Residual force

#### 4.6.2. Spreadability Study

The spreadability of the oleogels was performed texture analyser HD plus instrument (Stable Microsystems, Godalming, UK). The molten formulations were kept in a female cone and allowed to solidify at 25 °C for 90 min. The initial trigger force was set as 5 g. The 45° male perspex cone was allowed to penetrate the female cone at 0.5 mm/s, keeping a clearance of 5 mm from the base. After that, the male cone was brought back to its usual height, i.e., 30 mm from the surface.

### 4.7. Molecular Characterization

#### 4.7.1. Fourier Transform Infrared (FTIR) Spectroscopy

IR spectra of all the oleogels were studied using an FTIR spectrophotometer (Alpha-E; Bruker, Billerica, MA, USA) that was working in the attenuated total reflectance (ATR) mode. The oleogels were scanned within the wavenumber range 600–4000 cm^−1^, each with 25 scans. The spectral resolution of the instrument was 4 cm^−1^. The IR spectra of pure SFO, SFW, SPAN-80, and TWEEN-80 were also collected similarly.

#### 4.7.2. X-ray Diffraction Study

XRD of the oleogels was performed using an X-ray diffractometer (Model: Bruker D8 Advance, Austin, TX, USA) that was fitted with a Co-Kα radiation source (wavelength = 1.79 Å). The analysis was done at a voltage of 35 kV and a current of 25 mA. The X-ray scanning was carried out in the 2θ range from 5° to 50° and a scan rate of 5° 2θ/min. The XRD parameters of d-spacing (d) were calculated using Brag’s law (Equation (6)), and the crystallite size (D) was calculated using Debye–Scherrer equation (Equation (7)). Further lattice strain (ϵ) and dislocation density (δ) were calculated using Equations (8) and (9).

(7) λn =2dsinθ,

where λ = 1.79Å, i.e., the wavelength of the X-ray, n is an integer value, and θ is the diffraction angle.

(8) $$D=\frac{\lambda \;k}{\beta .\cos\theta },$$

where k is Scherrer constant, β is in radian and represents the full width at half maxima (FWHM) at a scattering angle 2θ.

(9) ϵ = β/4tanθ,

(10) $$\delta \;=\;\frac{1}{D^{2}}.$$

Here, dislocation density (δ) is represented in lines/m^2^.

### 4.8. Thermal Analysis

#### 4.8.1. Gelation Kinetics

The gelation kinetics of oleogels were studied with a lab-built temperature sensor system. For the experiment, 10 g of molten oleogels were transferred in a glass bottle (volume: 15 mL). The oleogel containing glass bottles were heated at 80 °C in a water bath. The bottles were then connected to the temperature sensor system and placed in the refrigerated water bath at 5 °C. Gelation of the formed oleogel was recorded for 90 min after the molten solution achieved a temperature of 50 °C through a time v/s temperature plot.

#### 4.8.2. Differential Scanning Calorimetry (DSC) Analysis

The thermal properties of the oleogels were inspected using a differential scanning calorimeter (200 F3 DSC, Maia, Netzsch, Burlington, MA, USA) at a thermal scan of 5 °C/min for their melting and crystallization capabilities. For this study, ~10 mg of oleogel was weighed in an aluminium pan and sealed using a pierced lid. A single program was run for each oleogel where the melting profiles from 0 °C to 100 °C and a cooling profile from 100 °C to 0 °C were recorded. In between each run, the oleogels were maintained at 100 °C for 5 min.

### 4.9. Drug Release Study

In-vitro drug release was performed through dissolution apparatus (Model; DA8000, Labindia Instruments Pvt. Ltd.; Mumbai; India). The dissolution apparatus consists of 6 stations, and the study was conducted by USP type I (Basket Type). A 20 g formulation consisting of 5 mg/g (*w*/*w*) curcumin was prepared through the trituration method for each oleogel. Then, 1.0 g of curcumin-loaded oleogel were placed into the basket and the medium for the release was 500 mL of double-distilled water (pH 6.8) at 37 ± 0.5 °C. The basket was fixed to 50 rpm. At a regular interval of time, 5 mL of the sample was withdrawn, and the same was replaced with fresh double-distilled water. The samples were analysed for the curcumin content at 425 nm using a UV-visible spectrophotometer (Model: Shimadzu 1800, Japan). The data was obtained in triplicates. The time-dependent curcumin release profile was plotted as cumulative percent drug release (CPDR).

### 4.10. Statistical Analyses

The data analysis in the various studies was performed in triplicate and therefore has been reported as mean ± standard deviation. The student *t*-test was done through IBM SPSS Statistics (Version 20) to confirm the significant differences (*p* < 0.05).

## Figures and Tables

**Table 1:** Parameters of Weichert’s modelling of oleogel formulation.

	P_0_	P_1_	τ_1_ (s)	P_2_	τ_2_ (s)	R^2^
Control	0.156 ± 0.01 ^efghi^	0.114 ± 0.01 ^ce^	13.298 ± 0.10 ^b^	0.726 ± 0.01 ^a^	0.314 ± 0.01 ^a^	0.99
S1	0.161 ± 0.01 ^gh^	0.163 ± 0.07 ^abcd^	12.860 ± 0.07 ^cdefh^	0.713 ± 0.01 ^abc^	0.247 ± 0.01 ^g^	0.99
S3	0.157 ± 0.01 ^h^	0.140 ± 0.01 ^a^	12.770 ± 0.17 ^efgh^	0.697 ± 0.01 ^bc^	0.312 ± 0.01 ^a^	0.99
S5	0.181 ± 0.01 ^cdei^	0.125 ± 0.01 ^abe^	13.910 ± 0.02 ^a^	0.697 ± 0.02 ^abc^	0.255 ± 0.01 ^fg^	0.99
S10	0.180 ± 0.02 ^defghi^	0.111 ± 0.01 ^de^	13.733 ± 1.29 ^abcdefgh^	0.711 ± 0.02 ^abc^	0.281 ± 0.03 ^abefg^	0.99
T1	0.187 ± 0.01 ^bcd^	0.115 ± 0.02 ^ab^	12.680 ± 0.09 ^g^	0.697 ± 0.01 ^c^	0.263 ± 0.01 ^ef^	0.99
T3	0.246 ± 0.01 ^a^	0.132 ± 0.01 ^ae^	12.600 ± 0.17 ^hg^	0.623 ± 0.01 ^d^	0.287 ± 0.01 ^b^	0.99
T5	0.163 ± 0.01 ^fgh^	0.116 ± 0.01 ^abe^	12.770 ± 0.01 ^fgh^	0.723 ± 0.03 ^abc^	0.271 ± 0.02 ^bdefg^	0.99
T10	0.172 ± 0.01 ^efgi^	0.116 ± 0.01 ^be^	12.853 ± 0.03 ^deh^	0.717 ± 0.02 ^abc^	0.273 ± 0.02 ^bcefg^	0.99

Superscripts with different alphabets in the same column represents statistically significant (*p* ≤ 0.05) values.

**Table 2:** XRD parameters obtained from deconvoluted peaks.

Formulations	Peak	Peak Position (°2θ)	FWHM (°2θ)	d-Spacing (Å)	Crystallite Size (nm)	Lattice Strain	Dislocation Density (δ) × 10^17^ Lines/m^2^
Control	1	18.95	4.00	5.43	2.44	0.10	0.17
	2	22.51	4.49	4.58	2.19	0.10	0.21
	3	25.05	0.43	4.13	23.11	0.01	0.00
	4	25.05	6.56	4.13	1.51	0.13	0.44
	5	27.86	0.43	3.72	23.24	0.01	0.00
Average			3.18	4.40	10.50	0.07	0.16
S1	1	18.88	3.55	5.45	2.75	0.09	0.13
	2	22.54	4.66	4.58	2.11	0.10	0.22
	3	25.15	0.41	4.11	24.03	0.01	0.00
	4	25.15	6.60	4.11	1.50	0.13	0.44
	5	27.88	0.40	3.71	24.83	0.01	0.00
Average			3.12	4.39	11.04	0.07	0.16
S3	1	15.93	2.31	6.46	4.22	0.07	0.06
	2	23.02	5.47	4.48	1.80	0.12	0.31
	3	25.18	0.41	4.10	24.29	0.01	0.00
	4	27.37	2.86	3.78	3.46	0.05	0.08
	5	27.97	0.44	3.70	22.81	0.01	0.00
Average			2.30	4.51	11.32	0.05	0.09
S5	1	18.47	3.48	5.57	2.81	0.09	0.13
	2	22.63	5.01	4.56	1.96	0.11	0.26
	3	25.15	0.41	4.11	24.12	0.01	0.00
	4	26.27	5.15	3.94	1.92	0.10	0.27
	5	27.89	0.43	3.71	23.32	0.01	0.00
Average			2.90	4.38	10.83	0.06	0.13
S10	1	18.30	3.25	5.62	3.00	0.09	0.11
	2	22.56	4.92	4.57	2.00	0.11	0.25
	3	24.80	7.57	4.17	1.30	0.15	0.59
	4	25.18	0.41	4.10	24.30	0.01	0.00
	5	27.95	0.41	3.70	24.01	0.01	0.00
Average			3.31	4.43	10.92	0.07	0.19
T1	1	18.89	1.82	5.45	5.35	0.05	0.03
	2	23.03	5.44	4.48	1.81	0.12	0.31
	3	25.20	0.43	4.10	23.10	0.01	0.00
	4	26.14	0.96	3.95	10.30	0.02	0.01
	5	27.93	1.85	3.71	5.38	0.03	0.03
Average			2.10	4.34	9.19	0.04	0.08
T3	1	17.46	0.50	5.89	19.62	0.01	0.00
	2	22.79	5.64	4.53	1.74	0.12	0.33
	3	25.14	0.42	4.11	23.35	0.01	0.00
	4	26.96	2.50	3.84	3.96	0.05	0.06
	5	27.95	0.45	3.70	21.93	0.01	0.00
Average			1.90	4.41	14.12	0.04	0.08
T5	1	22.53	7.31	4.58	1.34	0.16	0.56
	2	22.94	3.88	4.50	2.53	0.08	0.16
	3	25.13	0.43	4.11	23.13	0.01	0.00
	4	27.09	4.86	3.82	2.04	0.09	0.24
	5	27.92	0.41	3.71	24.53	0.01	0.00
Average			2.39	4.03	13.06	0.05	0.10
T10	1	19.13	3.95	5.38	2.47	0.10	0.16
	2	22.47	4.41	4.59	2.23	0.10	0.20
	3	25.09	0.41	4.12	24.14	0.01	0.00
	4	25.36	5.37	4.07	1.84	0.10	0.30
	5	27.88	0.39	3.71	25.58	0.01	0.00
Average			2.91	4.38	11.25	0.06	0.13

**Table 3:** Parameters obtained through exponential decay modelling of gelation kinetics of formulation.

Formulations	Temperature vs. Time		Exponential Decay Model	
	Onset of Secondary Crystallization (s)	Time to Reach Thermal Equilibrium (s)	Initial Rate of Crystallization (k) (°C/ms)	Initial Temperature of Crystallization (a) (°C)
Control	621	1968	2.88	50
S1	733	1995	2.65	50
S3	1075	2008	1.44	50
S5	1084	2348	1.12	50
S10	908	2092	1.64	50
T1	1138	2663	1.11	50
T3	1140	2981	1.01	50
T5	820	2134	0.9	50
T10	1223	2706	1.02	50

**Table 4:** Parameters of Peppas Sahlin (PS) model of drug release.

Sample	K_d_	K_r_	K_d_/K_r_	m	R^2^
Control	3.33 ± 0.01 ^bc^	0.52 ± 0.01 ^cd^	6.40 ± 0.02 ^abg^	0.32 ± 0.01 ^d^	0.99
S1	2.37 ± 0.06 ^d^	0.53 ± 0.02 ^bcd^	4.49 ± 0.09 ^c^	0.24 ± 0.01 ^ghi^	0.99
S3	1.78 ± 0.02 ^g^	0.48 ± 0.01 ^a^	3.72 ± 0.01 ^d^	0.25 ± 0.01 ^fgh^	0.99
S5	2.24 ± 0.05 ^ef^	0.66 ± 0.03 ^e^	3.41 ± 0.10 ^e^	0.23 ± 0.01 ^hi^	0.99
S10	1.03 ± 0.06 ^h^	0.51 ± 0.01 ^d^	2.03 ± 0.09 ^f^	0.29 ± 0.01 ^e^	0.99
T1	1.86 ± 0.28 ^fg^	0.23 ± 0.01 ^hi^	8.02 ± 1.26 ^b^	0.33 ± 0.01 ^bce^	0.99
T3	0.52 ± 0.02 ^i^	0.39 ± 0.01 ^f^	1.33 ± 0.06 ^gd^	0.50 ± 0.01 ^a^	0.99
T5	3.29 ± 0.57 ^cde^	0.22 ± 0.02 ^e^	15.10 ± 3.67 ^ab^	0.33 ± 0.01 ^cd^	0.99
T10	6.72 ± 0.21 ^a^	0.32 ± 0.01 ^g^	20.94 ± 0.58 ^a^	0.23 ± 0.01 ^i^	0.99

Superscripts with different alphabets in the same column represents statistically significant (*p* ≤ 0.05) values.

**Table 5:** Composition of prepared formulations.

Samples	SFW (g)	SO (g)	Emulsifier Stock (g)
Control	1.0	19.0	0.0
S1	1.0	18.0	1.0
S3	1.0	16.0	3.0
S5	1.0	14.0	5.0
S10	1.0	9.0	10.0
T1	1.0	18.0	1.0
T3	1.0	16.0	3.0
T5	1.0	14.0	5.0
T10	1.0	9.0	10.0

## Data Availability

The data will be available from the corresponding authors on request.

## Supplementary Materials

The following are available online at https://www.mdpi.com/article/10.3390/gels7030133/s1. Table S1: Values of absolute color difference (ΔE). Figure S1: Spreadability profile of a) SPAN-80 formulation b) TWEEN-80 formulation. Table S2: Parameters of spreadability test of oleogel. Figure S2: FTIR spectra of raw components and control oleogel. Figure S3: FTIR spectra all the formulations. Table S3: Thermal properties of oleogels.

## References

[1] Scharfe M., Flöter E. (2020). Oleogelation: From Scientific Feasibility to Applicability in Food Products. Eur. J. Lipid Sci. Technol..

[2] Mozaffarian D., Ludwig D. (2015). The 2015 US Dietary Guidelines: Lifting the Ban on Total Dietary Fat. JAMA.

[3] Pușcaș A., Mureșan V., Socaciu C., Muste S. (2020). Oleogels in Food: A Review of Current and Potential Applications. Foods.

[4] Dassanayake L.S.K., Kodali D.R., Ueno S. (2011). Formation of oleogels based on edible lipid materials. Curr. Opin. Colloid Interface Sci..

[5] Delbecq F., Nguyen R., Van Hecke E., Len C. (2019). Design and physicochemical properties of long and stiff fatty low molecular weight oleogelators. J. Mol. Liq..

[6] Winkler-Moser J.K., Anderson J., Felker F.C., Hwang H. (2019). Physical Properties of Beeswax, Sunflower Wax, and Candelilla Wax Mixtures and Oleogels. J. Am. Oil Chem. Soc..

[7] Bin Sintang M.D., Danthine S., Tavernier I., Van de Walle D., Doan C.D., Muhammad D.R.A., Rimaux T., Dewettinck K. (2021). Polymer coated fat crystals as oil structuring agents: Fabrication and oil-structuring properties. Food Hydrocoll..

[8] Okuro P.K., Tavernier I., Bin Sintang M.D., Skirtach A.G., Vicente A.A., Dewettinck K., Cunha R.L. (2018). Synergistic interactions between lecithin and fruit wax in oleogel formation. Food Funct..

[9] Tavernier I., Doan C.D., Van Der Meeren P., Heyman B., Dewettinck K. (2018). The Potential of Waxes to Alter the Microstructural Properties of Emulsion-Templated Oleogels. Eur. J. Lipid Sci. Technol..

[10] Hwang H.-S., Kim S., Evans K., Koga C., Lee Y. (2015). Morphology and networks of sunflower wax crystals in soybean oil organogel. Food Struct..

[11] Hwang H.-S., Singh M., Winkler-Moser J., Bakota E.L., Liu S.X. (2014). Preparation of Margarines from Organogels of Sunflower Wax and Vegetable Oils. J. Food Sci..

[12] Franco R. (2018). Sunflower Oil Functional Properties for Specialty Food. Nutr. Food Sci. Int. J..

[13] Oh I.K., Amoah C., Lim J., Jeong S., Lee S. (2017). Assessing the effectiveness of wax-based sunflower oil oleogels in cakes as a shortening replacer. LWT.

[14] Liu N., Lu Y., Zhang Y., Gao Y., Mao L. (2020). Surfactant addition to modify the structures of ethylcellulose oleogels for higher solubility and stability of curcumin. Int. J. Biol. Macromol..

[15] Ribeiro A.P.B., Masuchi M.H., Miyasaki E.K., Domingues M.A.F., Stroppa V.L.Z., De Oliveira G.M., Kieckbusch T.G. (2014). Crystallization modifiers in lipid systems. J. Food Sci. Technol..

[16] Uvanesh K., Nayak S.K., Sagiri S.S., Banerjee I., Ray S.S., Pal K. (2018). Effect of Non-Ionic Hydrophilic and Hydrophobic Surfactants on the Properties on the Stearate Oleogels. Nutraceuticals and Innovative Food Products for Healthy Living and Preventive Care.

[17] Fu X., Kong W., Zhang Y., Jiang L., Wang J., Lei J. (2015). Novel solid–solid phase change materials with biodegradable trihydroxy surfactants for thermal energy storage. RSC Adv..

[18] Yeh C.K.-J., Peng S.-L., Hsu I.-Y. (2002). Co-surfactant of ethoxylated sorbitan ester and sorbitan monooleate for enhanced flushing of tetrachloroethylene. Chemosphere.

[19] Fayaz G., Calligaris S., Nicoli M.C. (2019). Comparative Study on the Ability of Different Oleogelators to Structure Sunflower Oil. Food Biophys..

[20] Meng Z., Qi K., Guo Y., Wang Y., Liu Y. (2018). Effects of thickening agents on the formation and properties of edible oleogels based on hydroxypropyl methyl cellulose. Food Chem..

[21] Sagiri S.S., Rao K. (2020). Natural and bioderived molecular gelator–based oleogels and their applications. Biopolymer-Based Formulations: Biomedical and Food Applications.

[22] Pehlivanoglu H., Demirci M., Toker O.S. (2017). Rheological properties of wax oleogels rich in high oleic acid. Int. J. Food Prop..

[23] León K., Mery D., Pedreschi F., León J. (2006). Color measurement in L∗a∗b∗ units from RGB digital images. Food Res. Int..

[24] Youssef M., Barbut S. (2011). Fat reduction in comminuted meat products-effects of beef fat, regular and pre-emulsified canola oil. Meat Sci..

[25] Yam K.L., Papadakis S.E. (2004). A simple digital imaging method for measuring and analyzing color of food surfaces. J. Food Eng..

[26] Kupiec M., Zbikowska A., Marciniak-Lukasiak K., Kowalska M. (2020). Rapeseed Oil in New Application: Assessment of Structure of Oleogels Based on their Physicochemical Properties and Microscopic Observations. Agriculture.

[27] Rizzo G., Norton J., Norton I. (2015). Emulsifier effects on fat crystallisation. Food Struct..

[28] Maruyama J.M., Soares F.A.S.D.M., D’Agostinho N.R., Gonçalves M.I.A., Gioielli L.A., Da Silva R.C. (2014). Effects of Emulsifier Addition on the Crystallization and Melting Behavior of Palm Olein and Coconut Oil. J. Agric. Food Chem..

[29] Patel A.R. (2015). Alternative Routes to Oil Structuring.

[30] Cabrera S., Rojas J., Moreno A. (2020). Contribution in the Production of Healthier Food Products: The Fats of the Future. J. Food Nutr. Res..

[31] Terech P. (1996). Networks of surfactant-made physical organogels. Prog. Colloid Polym. Sci..

[32] ED C.O., Marangoni A.G. (2018). Oleogels. Edible Oleogels.

[33] Samateh S., Sagiri S., John G. (2018). Molecular Oleogels. Edible Oleogels.

[34] Kesselman E., Shimoni E. (2007). Imaging of Oil/Monoglyceride Networks by Polarizing Near-Field Scanning Optical Microscopy. Food Biophys..

[35] Palla C., de Vicente J., Carrín M.E., Gálvez-Ruiz M.J. (2019). Effects of cooling temperature profiles on the monoglycerides oleogel properties: A rheo-microscopy study. Food Res. Int..

[36] Blake I., Toro-Vazquez J.F., Hwang H.-S. (2018). Wax Oleogels. Edible Oleogels.

[37] Blake A.I., Co E.D., Marangoni A.G. (2014). Structure and Physical Properties of Plant Wax Crystal Networks and Their Relationship to Oil Binding Capacity. J. Am. Oil Chem. Soc..

[38] Bin Sintang M.D., Rimaux T., Van De Walle D., Dewettinck K., Patel A.R. (2016). Oil structuring properties of monoglycerides and phytosterols mixtures. Eur. J. Lipid Sci. Technol..

[39] Doan C.D., Tavernier I., Okuro P.K., Dewettinck K. (2018). Internal and external factors affecting the crystallization, gelation and applicability of wax-based oleogels in food industry. Innov. Food Sci. Emerg. Technol..

[40] Yamaue T., Doi M. (2005). The stress diffusion coupling in the swelling dynamics of cylindrical gels. J. Chem. Phys..

[41] Uvanesh K., Sagiri S.S., Senthilguru K., Pramanik K., Banerjee I., Anis A., Al-Zahrani S.M., Pal K. (2015). Effect of Span 60 on the Microstructure, Crystallization Kinetics, and Mechanical Properties of Stearic Acid Oleogels: An In-Depth Analysis. J. Food Sci..

[42] Meng Z., Guo Y., Wang Y., Liu Y. (2018). Oleogels from sodium stearoyl lactylate-based lamellar crystals: Structural characterization and bread application. Food Chem..

[43] Mohanan A., Tang Y.R., Nickerson M.T., Ghosh S. (2020). Oleogelation using pulse protein-stabilized foams and their potential as a baking ingredient. RSC Adv..

[44] Sagiri S.S., Kasiviswanathan U., Shaw G.S., Singh M., Anis A., Pal K. (2016). Effect of sorbitan monostearate concentration on the thermal, mechanical and drug release properties of oleogels. Korean J. Chem. Eng..

[45] Yadav I., Kasiviswanathan U., Soni C., Paul S.R., Nayak S.K., Sagiri S.S., Anis A., Pal K. (2017). Stearic Acid Modified Stearyl Alcohol Oleogel: Analysis of the Thermal, Mechanical and Drug Release Properties. J. Surfactants Deterg..

[46] Xu X., Liu B., Li Y. (2015). Experimental Studies on Viscoelasticity of Film Materials in Laminated Glass Sheets. SAE Int. J. Mater. Manuf..

[47] Kodela S.P., Pandey P.M., Nayak S.K., Uvanesh K., Anis A., Pal K. (2017). Novel agar–stearyl alcohol oleogel-based bigels as structured delivery vehicles. Int. J. Polym. Mater..

[48] Rohman A., Man Y.B.C. (2012). Quantification and Classification of Corn and Sunflower Oils as Adulterants in Olive Oil Using Chemometrics and FTIR Spectra. Sci. World J..

[49] Yi Y., Yao J., Xu W., Wang L.-M., Wang H.-X. (2019). Investigation on the quality diversity and quality-FTIR characteristic relationship of sunflower seed oils. RSC Adv..

[50] Missau J., Rocha J.D.G.D., Dotto G.L., Bertuol D., Ceron L.P., Tanabe E.H. (2018). Purification of crude wax using a filter medium modified with a nanofiber coating. Chem. Eng. Res. Des..

[51] Dubey P., Sharma P., Kumar V. (2017). FTIR and GC–MS spectral datasets of wax from Pinus roxburghii Sarg. needles biomass. Data Brief.

[52] Lin B.-J., Chen W.-H., Budzianowski W.M., Hsieh C.-T., Lin P.-H. (2016). Emulsification analysis of bio-oil and diesel under various combinations of emulsifiers. Appl. Energy.

[53] Bora M.M., Gogoi P., Deka D.C., Kakati D.K. (2014). Synthesis and characterization of yellow oleander (Thevetia peruviana) seed oil-based alkyd resin. Ind. Crop. Prod..

[54] Farooq A., Shafaghat H., Jae J., Jung S.-C., Park Y.-K. (2018). Enhanced stability of bio-oil and diesel fuel emulsion using Span 80 and Tween 60 emulsifiers. J. Environ. Manag..

[55] Ongpipattanakul B., Francoeur M.L., Potts R.O. (1994). Polymorphism in stratum corneum lipids. Biochim. Biophys. Acta (BBA)-Biomembr..

[56] Cameron D.G., Gudgin E.F., Mantsch H.H. (1981). Dependence of acyl chain packing of phospholipids on the head group and acyl chain length. Biochemistry.

[57] Das P., Qureshi D., Paul S., Mohanty B., Anis A., Verma S., Wilczyński S., Pal K. (2021). Effect of sorbitan monopalmitate on the polymorphic transitions and physicochemical properties of mango butter. Food Chem..

[58] Holey S.A., Sekhar K.P.C., Mishra S.S., Kanjilal S., Nayak R.R. (2020). Sunflower Wax-Based Oleogel Emulsions: Physicochemical Characterizations and Food Application. ACS Food Sci. Technol..

[59] Calligaris S., Arrighetti G., Barba L., Nicoli M.C. (2008). Phase Transition of Sunflower Oil as Affected by the Oxidation Level. J. Am. Oil Chem. Soc..

[60] Hoffmann H.D.M. (1987). Small: The physical chemistry of lipidis: From alkanes to phospholipids, Plenum Press, New York and London 1986. 672 Seiten, Preis: $89.50 + 20%, with contribution by Bryan M. Craven, Yvonne Lange, G. Graham Shipley and John Steiner. Ber. Bunsenges. Phys. Chem..

[61] Fats and Oils: Formulating and Processing for Applications, Third Edition—PDF Free Download. https://epdf.pub/fats-and-oils-formulating-and-processing-for-applications-third-edition.html.

[62] Tavernier I., Doan C.D., Van de Walle D., Danthine S., Rimaux T., Dewettinck K. (2017). Sequential crystallization of high and low melting waxes to improve oil structuring in wax-based oleogels. RSC Adv..

[63] Sun P., Xia B., Ni Z.-J., Wang Y., Elam E., Thakur K., Ma Y.-L., Wei Z.-J. (2021). Characterization of functional chocolate formulated using oleogels derived from β-sitosterol with γ-oryzanol/lecithin/stearic acid. Food Chem..

[64] Hasda A.M., Vuppaladadium S.S.R., Qureshi D., Prasad G., Mohanty B., Banerjee I., Shaikh H., Anis A., Sarkar P., Pal K. (2020). Graphene oxide reinforced nanocomposite oleogels improves corneal permeation of drugs. J. Drug Deliv. Sci. Technol..

[65] Chaves K., Silva T.J., Domingues M.A.F., Barrera-Arellano D., Ribeiro A.P.B. (2019). Conventional and Unconventional Crystallization Mechanisms. Crystal Growth.

[66] Liu C., Zheng Z., Meng Z., Chai X., Cao C., Liu Y. (2019). Beeswax and carnauba wax modulate the crystallization behavior of palm kernel stearin. LWT.

[67] Sagiri S., Sharma V., Basak P., Pal K. (2014). Mango Butter Emulsion Gels as Cocoa Butter Equivalents: Physical, Thermal, and Mechanical Analyses. J. Agric. Food Chem..

[68] Öğütcü M., Yılmaz E. (2014). Characterization of Hazelnut Oil Oleogels Prepared with Sunflower and Carnauba Waxes. Int. J. Food Prop..

[69] Yılmaz E., Öğütcü M. (2014). Comparative Analysis of Olive Oil Organogels Containing Beeswax and Sunflower Wax with Breakfast Margarine. J. Food Sci..

[70] Ferguson R.H., Lutton E.S. (1941). The Polymorphic Forms or Phases of Triglyceride Fats. Chem. Rev..

[71] Hondoh H., Ueno S. (2016). Polymorphism of edible fat crystals. Prog. Cryst. Growth Charact. Mater..

[72] Ghotra B.S., Dyal S.D., Narine S.S. (2002). Lipid shortenings: A review. Food Res. Int..

[73] Martini S., Añón M.C. (2003). Crystallization of sunflower oil waxes. J. Am. Oil Chem. Soc..

[74] Hani U. (2014). Solubility Enhancement and Delivery Systems of Curcumin a Herbal Medicine: A Review. Curr. Drug Deliv..

[75] Peppas N.A., Sahlin J.J. (1989). A simple equation for the description of solute release. III. Coupling of diffusion and relaxation. Int. J. Pharm..

[76] Ghosal K., Chandra A., Rajabalaya R., Chakraborty S., Nanda A. (2012). Mathematical modeling of drug release profiles for modified hydrophobic HPMC based gels. Die Pharm..

[77] Freire M.C.L.C., Alexandrino F., Marcelino H.R., Picciani P.H.D.S., Silva K.G.D.H.E., Genre J., De Oliveira A.G., Egito E.S.T.D. (2017). Understanding Drug Release Data through Thermodynamic Analysis. Materials.

[78] Abdollahi M., Goli S.A.H., Soltanizadeh N. (2019). Physicochemical Properties of Foam-Templated Oleogel Based on Gelatin and Xanthan Gum. Eur. J. Lipid Sci. Technol..

[79] Jain A., Pradhan B.K., Mahapatra P., Ray S.S., Chakravarty S., Pal K. (2020). Development of a low-cost food color monitoring system. Color Res. Appl..

