# Supplementary material for: Effect of Biodegradable Hydrophilic and Hydrophobic Emulsifiers on the Oleogels Containing Sunflower Wax and Sunflower Oil
Source: Gels. 2021 Sep 7;7(3):133. doi: 10.3390/gels7030133 (PMC8482198; doi:10.3390/gels7030133)
Supplement: Supplementary file 1 [file gels-07-00133-s001.zip › gels-1301104-supplementary.pdf]

gels

Article

Effect of Biodegradable Hydrophilic and Hydrophobic Emulsifiers on the Oleogels Containing Sunflower Wax and Sunflower Oil

Deepti Bharti [1], Doman Kim [2], Miguel Ângelo Cerqueira [3], Biswaranjan Mohanty [4], SK. Habibullah [4], Indranil Banerjee [5] and Kunal Pal [1,*]

Supplementary file

1. Colorimetric analysis

Table S1. Values of absolute color difference (ΔE).

Sample name	ΔE
S1	6.27 ± 0.59
S3	6.77 ± 1.05
S5	4.40 ± 0.29
S10	0.51 ± 0.50
T1	1.12 ± 0.09
T3	8.90 ± 0.79
T5	6.60 ± 0.87
T10	8.20 5.08

2. Mechanical Study

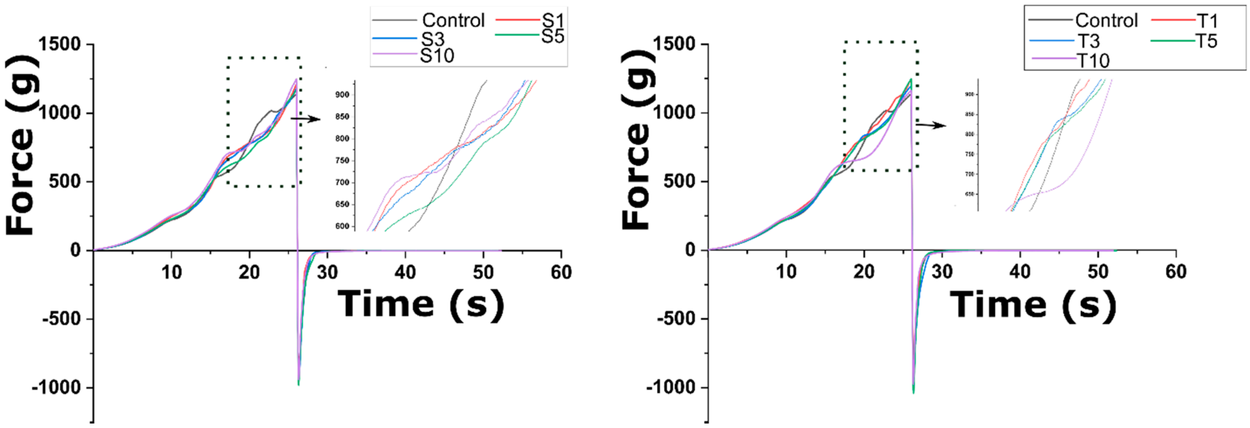

Figure S1. Spreadability profile of a) SPAN-80 formulation b)TWEEN-80 formulation.

Table S2. Parameters of spreadability test of oleogel.

Samples	Firmness(g) (F_0)	Work of shear(g-mm) (C_0)	Stickiness(g) (S_0)	Work of adhesion(g-mm) (A_0)
Control	1140.47±77.47	11468.39±325.88	-876.72±84.96	-715.63±4.59
S1	1123.11±92.63	10285.46±1408.87	-807.72±4.86	-633.745±88.45
S3	1182.91±32.30	11430.46±793.75	-930.82±113.80	-766.04±220.67
S5	1166.30±128.60	11144.55±1182.20	-988.91±143.84	-804.60±167.19
S10	1249.38±187.93	11938.27±1332.53	-943.84±162.30	-614.38±39.74
T1	1247.74±293.06	12089.96±2312.73	-970.37±345.57	-732.29±19.60
T3	1194.51±75.85	11594.57±389.57	-1024.05±149.28	-801.68±119.23
T5	1249.82±123.32	11669.31±557.52	-1041.922±138.13	-735.96±32.44
T10	1164.41±29.25	11166.34±221.46	-977.15±27.14	-665.56±48.46

3. FTIR analysis

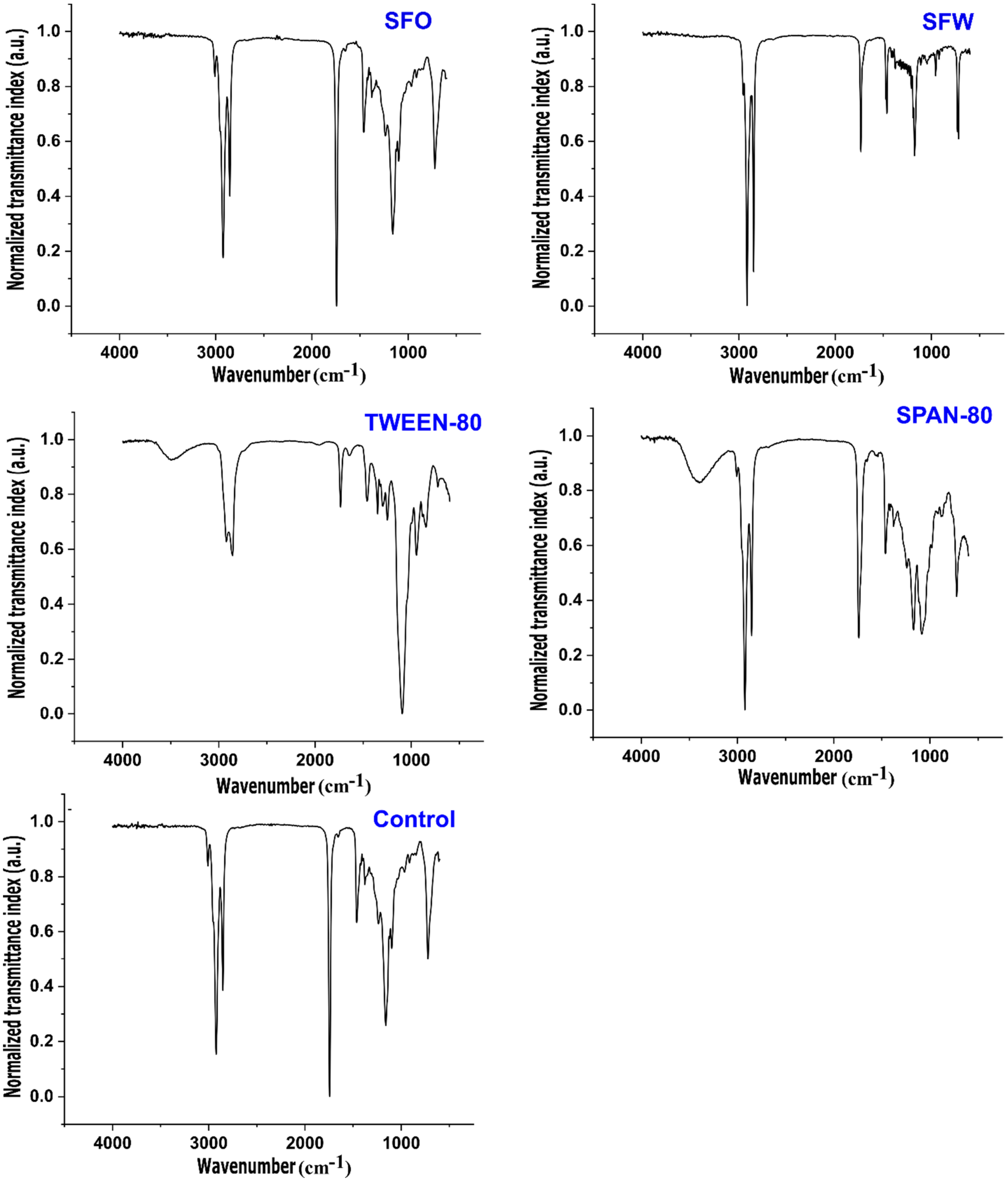

Figure S2. FTIR spectra of raw components and control oleogel.

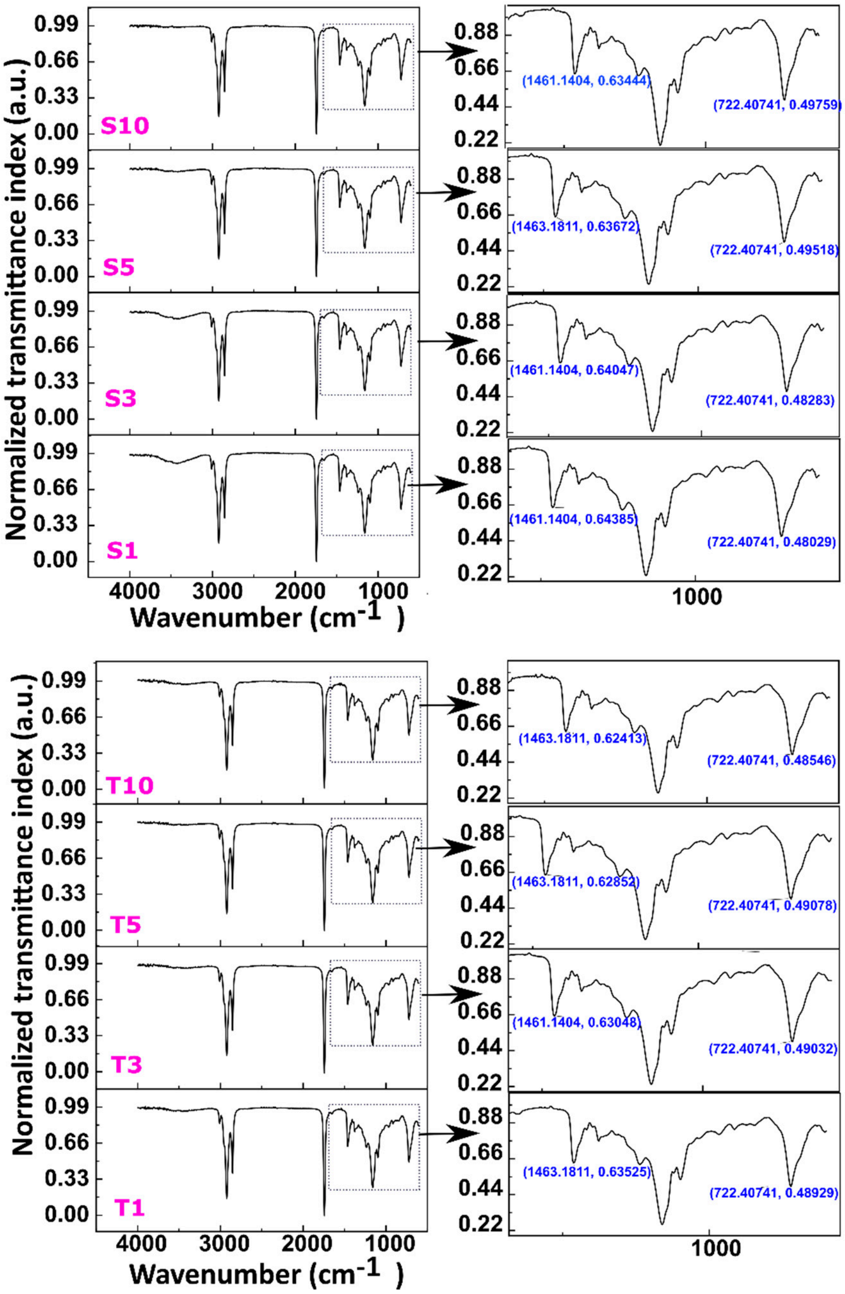

Figure S3. FTIR spectra all the formulations.

4. DSC analysis

Table S3. Thermal properties of oleogels.

Formulations	Peaks	Temperature (°C)	Area	Onset(°C)	Peak (°C)
**Control**	Peak 1	62.019	0.3714	63.43	60.93
	Peak 2	65.905	0.084		
**S1**	Peak 1	60.25	0.318	63.37	60.87
	Peak 2	64.490	0.181		
**S3**	Peak 1	64.105	0.422	63.40	60.89
	Peak 2	66.544	0.054		
**S5**	Peak 1	60.481	0.320	63.41	59.665
	Peak 2	65.110	0.131		
**S10**	Peak 1	61.547	0.370	63.41	60.918
	Peak 2	65.447	0.113		
**T1**	Peak 1	60.676	0.442	63.41	60.911
	Peak 2	64.939	0.132		
**T3**	Peak 1	63.320	0.085	62.16	59.659
	Peak 2	66.088	0.079		
**T5**	Peak 1	60.459	0.070	63.41	59.665
	Peak 2	67.202	0.187		
**T10**	Peak 1	64.205	0.416	63.41	59.663
	Peak 2	66.280	0.050		
